# *De novo* transcriptomic assembly and profiling of *Rigidoporus microporus* during saprotrophic growth on rubber wood

**DOI:** 10.1186/s12864-016-2574-9

**Published:** 2016-03-15

**Authors:** Abbot O. Oghenekaro, Tommaso Raffaello, Andriy Kovalchuk, Fred O. Asiegbu

**Affiliations:** Department of Forest Sciences, University of Helsinki, P.O. Box 27, 00014 Helsinki, Finland

**Keywords:** *Rigidoporus microporus*, *Hevea brasiliensis*, RNA-Seq, *De-novo* assembly, Transcriptome, White rot fungi, Lignocellulose, Natural rubber

## Abstract

**Background:**

The basidiomycete *Rigidoporus microporus* is a fungus that causes the white rot disease of the tropical rubber tree, *Hevea brasiliensis*, the major source of commercial natural rubber. Besides its lifestyle as a pathogen, the fungus is known to switch to saprotrophic growth on wood with the ability to degrade both lignin and cellulose. There is almost no genomic or transcriptomic information on the saprotrophic abilities of this fungus. In this study, we present the fungal transcriptomic profiles during saprotrophic growth on rubber wood.

**Results:**

A total of 266.6 million RNA-Seq reads were generated from six libraries of the fungus growing either on rubber wood or without wood. *De novo* assembly produced 34, 518 unigenes with an average length of 2179 bp. Annotation of unigenes using public databases; GenBank, Swiss-Prot, Kyoto Encyclopedia of Genes and Genomes (KEGG), Cluster of Orthologous Groups (COG) and Gene Ontology (GO) produced 25, 880 annotated unigenes. Transcriptomic profiling analysis revealed that the fungus expressed over 300 genes encoding lignocellulolytic enzymes. Among these, 175 genes were up-regulated in rubber wood. These include three members of the glycoside hydrolase family 43, as well as various glycosyl transferases, carbohydrate esterases and polysaccharide lyases. A large number of oxidoreductases which includes nine manganese peroxidases were also significantly up-regulated in rubber wood. Several genes involved in fatty acid metabolism and degradation as well as natural rubber degradation were expressed in the transcriptome. Four genes (acyl-CoA synthetase, enoyl-CoA hydratase, 3-hydroxyacyl-CoA dehydrogenase and acyl-CoA acetyltransferase) potentially involved in rubber latex degradation pathway were also induced. A number of ATP binding cassette (ABC) transporters and hydrophobin genes were significantly expressed in the transcriptome during saprotrophic growth. Some genes related to energy metabolism were also induced.

**Conclusions:**

The analysed data gives an insight into the activation of lignocellulose breakdown machinery of *R. microporus*. This study also revealed genes with relevance in antibiotic metabolism (e.g. cephalosporin esterase) as well as those with potential applications in fatty acid degradation. This is the first study on the transcriptomic analysis of *R. microporus* on rubber wood and should serve as a pioneering resource for future studies of the fungus at the genomic or transcriptomic level.

**Electronic supplementary material:**

The online version of this article (doi:10.1186/s12864-016-2574-9) contains supplementary material, which is available to authorized users.

## Background

The white rot fungus *Rigidoporus microporus* (Polyporales, Basidiomycota) syn. *Rigidoporus lignosus* is the most destructive root pathogen of the tropical rubber tree, *Hevea brasiliensis* Muell. Arg, which is the major source of natural rubber [1]. It is an economically important pathogen of *H. brasiliensis* with yearly economic losses of millions of dollars in the tropics. The control and management of the white rot disease of rubber in most tropical countries have been hampered due to limited knowledge of the population genetics of the different isolates [2], as well as molecular basis of virulence mechanisms. Additionally, members of the white rot basidiomycota fungi are known to play major roles in nutrient and carbon cycling in temperate and tropical forests [3]. It is therefore expected that the *Rigidoporus* group will harbor a repertoire of a wide range of useful enzymes important for lignocellulose degradation with potential applications in bioenergy processing and utilization. However, there is presently no genomic or transcriptome resources available for any species within the genus, *Rigidoporus*.

In nature, the fungus infects over forty other tropical tree species including *Tectonia grandis*, *Artocarpus nobilis, Theobroma cacao* and *Cocos nucifera* [4–6], but the pathogen is a problem mainly in rubber plantations in Asia and Africa. The pathogen was a major problem on 43 % of *H. brasiliensis* plantations in a survey conducted in Malaysia in 1993 [7]. In Nigeria, *R. microporus* is responsible for 96 % of incidences of root diseases in rubber plantations [8].

The fungus produces rhizomorphs which can grow several meters in the soil and attach to wood debris. Above ground symptoms are only visible once the roots are completely damaged. The rhizomorphs remain in the soil after the death of trees and may serve as source of inoculum for infecting other trees or continue its survival by obtaining nutrients from dead wood [2, 9].

There is a high density of rhizomorphs and mycelia of the fungus in the soil of *H. brasiliensis* infected natural forests and plantations [10] indicating its capacity for a saprotrophic lifestyle. Besides being a serious pathogen, *R. microporus* is a typical white rot basidiomycete with the potential to degrade lignin and cellulose components of wood.

To obtain an overall view of all the processes that occur during fungal growth as well as during wood degradation, it is necessary to identify as many as possible genes that are expressed during the saprotrophic colonization. The use of high-throughput DNA sequencing has facilitated the characterization and identification of phytopathogenic fungi genes expressed during developmental stages or fungal pathogenicity [11]. RNA-Seq technology applied in this study detects novel genes as well as provides information about previously uncharacterized genes. Next-generation sequencing technologies have led to the generation of huge genomic and transcriptomic data that have improved our understanding of wood decay by white rot basidiomycetes. This revolution has evolved from single genome sequencing to large scale basidiomycete dual [12–14] and multiple [15–17] genome and transcriptome comparative analysis. The model white rot fungus, *Phanerochaete chrysosporium* genome and transcriptome has been studied in detail, revealing a rich repertoire of lignocellulose degrading genes [18, 19]. Other white rot species with transcriptomic profile information on growth on various carbohydrate sources include *Fomitiporia mediterranea* [20] and *Pycnoporus cinnabarinus* [21]. Genome and transcriptome of the white rot fungi, *Phlebiopsis gigantea* with resin and fatty acid degradation potential has also been studied [22]. On the other hand, there are very few studies on transcriptomic information regarding wood-decay basidiomycota with established parasitic and saprotrophic lifestyles. Genome and microarray transcriptome studies of the conifer root and butt pathogen, *Heterobasidion annosum* sensu lato (s.l) on pine wood revealed a plethora of glycoside hydrolases, multi copper oxidases and manganese peroxidase enzymes implicated in lignocellulose degradation [23, 24].

A search for *R. microporus* in the National Center for Biotechnology Information (NCBI) resource revealed no information relative to EST (Expressed sequence tag), Unigene and Gene, while there are only 36 protein sequences deposited (as of September, 2015). Genomic information related to the major pathogen host, *H. brasiliensis* has recently received more attention with the release of the draft genome sequence of the tree [25]. However, there is almost no information on the role of *R. microporus* during its saprotrophic lifestyle at the genomic and transcriptomic level. Additionally, most *Rigidoporus* species belong to the Meripilus clade of the Polyporales, one of the orders of Agaricomycetes. The transcriptome sequence would also contribute to further enrich the power of comparative genomics information in this basidiomycete group. Furthermore, the primary importance of the *Rigidoporus* transcriptome resource is partly due to the negative impact of this pathogen to productivity of tropical rubber tree in several parts of the world. The economic loss is enormous not only in terms of wood production but also on indirect impacts on global latex production on an important raw material for automobile and airplane tyre production.

The objectives of this study were; (1) to study the transcript profiles of genes expressed during saprotrophic growth of *R. microporus* on *H. brasiliensis* (2) to get an insight on the potential ability of the fungus to degrade natural rubber latex produced by the host and (3) to provide genetic resources that would facilitate further research at the molecular and genetic levels of the lifestyle of this fungus. As there is presently no genomic data available for this fungus, we performed RNA-Seq *de novo* assembly and compared the transcriptomes of the fungus grown on nutrient media with and without rubber wood. The results generated in this study would provide insights on the genes utilized by this fungus for lignocellulose degradation of rubber wood and also serve as an important resource for future studies on this economically important pathogen.

## Results

### Sequencing of the transcriptome

**Fig. 1:**
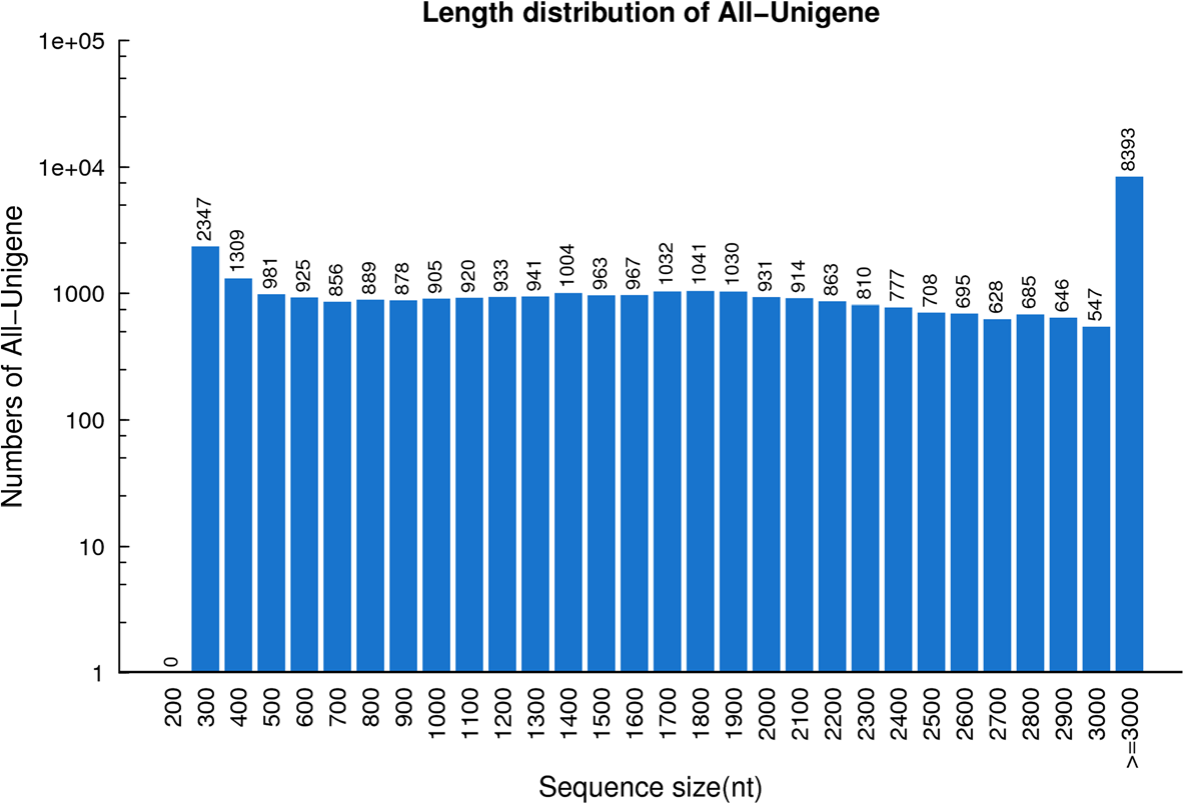
Length distribution of assembled *R. microporus* unigenes

The transcriptome of *R. microporus* was sequenced and *de novo* assembled since there are no genomic data available for the fungus. In order to capture a large number of transcripts, three replicates of the two conditions [W and C (W1, W2, W3, C1, C2, C3)] were sequenced separately. Each of the six samples produced over 40 million raw reads of single read length of 90 bp, resulting in a total of 266.6 million reads (Table 1). Clean reads [251.1 million (94.2 % of raw reads)] and clean nucleotides (22.6 billion) were obtained for assembly after quality control (Table 1). Assembly was carried out using the sequence clustering program, Trinity. Reads were assembled into 34,518 unigenes with a mean length of 2179 bp. Unigenes with length ≥ 3000 bp represent the highest number of assemblies (Fig. 1). Distinct clusters (26,447) represents cluster unigenes; the same cluster contains similar unigenes (>70 % similarity). Distinct singletons (8701) represent unigenes from a single gene (Table 2). Further sequencing quality control was done by mapping the clean reads to the assembled unigenes. Mapping results show a high mapping coverage (>95 %) for all samples (Additional file 1: Table S1).

**Table 1:** *R. microporus* transcriptome sequencing summary

Samples	Total raw reads	Total clean reads	Total clean nucleotides
W1	42,119,460	39,670,980	3,570,388,200
W2	43,022,126	40,551,694	3,649,652,460
W3	44,834,988	42,350,576	3,811,551,840
C1	40,064,424	37,800,314	3,402,028,260
C2	48,089,954	45,157,482	4,064,173,380
C3	48,451,512	45,603,760	4,104,338,400
All	266,582,464	251,134,806	22,602,132,540

**Table 2:** *R. microporus* transcriptome assembly results for Unigenes

Sample	Total number	Total length (nt)	Mean length (nt)	Distinct clusters	Distinct singletons
W1	28,191	44,113,047	1565	15,574	12,617
W2	29,990	52,269,204	1743	17,865	12,125
W3	36,725	59,724,821	1626	21,815	14,910
C1	20,472	29,140,707	1423	7781	12,691
C2	21,976	32,638,908	1485	9257	12,719
C3	24,026	38,235,592	1591	11,787	12,239
All	34,518	75,231,694	2179	26,447	8071

### Annotation and characteristics of the transcriptome

**Fig. 2:**
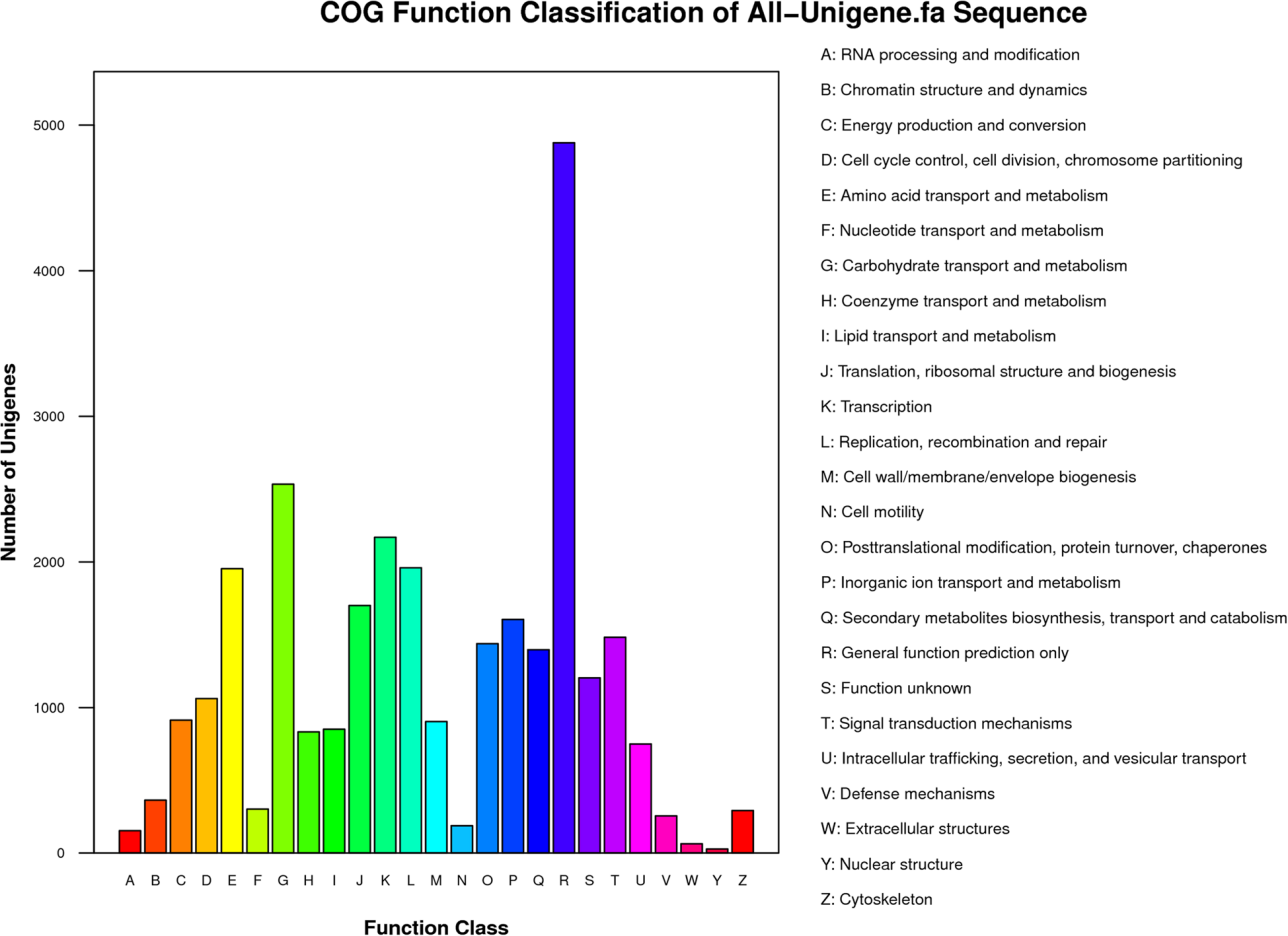
COG functional categories of *R. microporus* unigenes

**Fig. 3:**
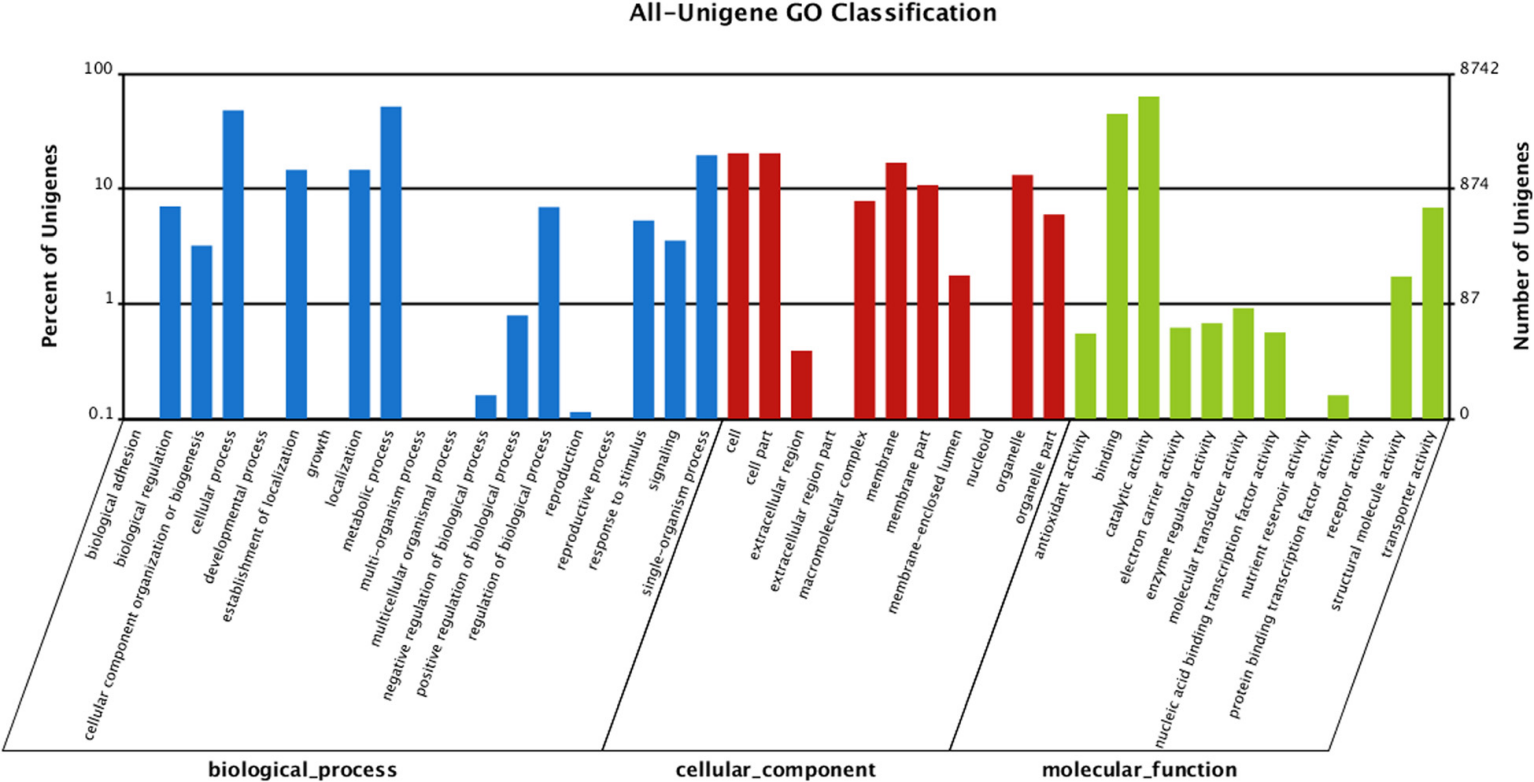
Gene Ontology classification of *R. microporus* transcriptome into biological process, cellular component and molecular function

Functional annotation of the unigenes was done by first aligning by Blastx (cut-off E-value < 10^−5^) to protein databases in the following order of priority: NR (GenBank), Swiss-Prot, Kyoto Encyclopedia of Genes and Genomes (KEGG), Cluster of Orthologous Groups (COG) and Gene Ontology (GO). The coding regions of unigenes were predicted based on the proteins with the highest rank in blast results. Protein coding prediction using Blastx produced 26,663 unigenes with predicted open reading frame (ORF). Unigenes that cannot align to any database were scanned by ESTScan to provide sequence direction of the predicted coding region. A total of 25,880 (74.98 %) of 34, 518 unigenes were functionally annotated (Additional file 2: Table S2). The complete list of number of annotated unigenes from public databases is shown in Additional file 3: Table S3. The sequence homology of *R. microporus* transcriptome against NR NCBI database is shown in Additional file 4: Figure S1. The *R. microporus* transcriptome showed a very strong match (51.6 % of hits) with *F. mediterranea* genome. Blastx results were used to classify unigenes and determine functional annotation for the unigenes in COG and GO. In the COG classification, the unigenes were divided into 25 functional groups with the ‘General function’ cluster representing the largest group (Fig. 2). The GO classification separated the unigenes into 42 functional groups representing biological process, cellular component and molecular function ontologies (Fig. 3).

**Fig. 4:**
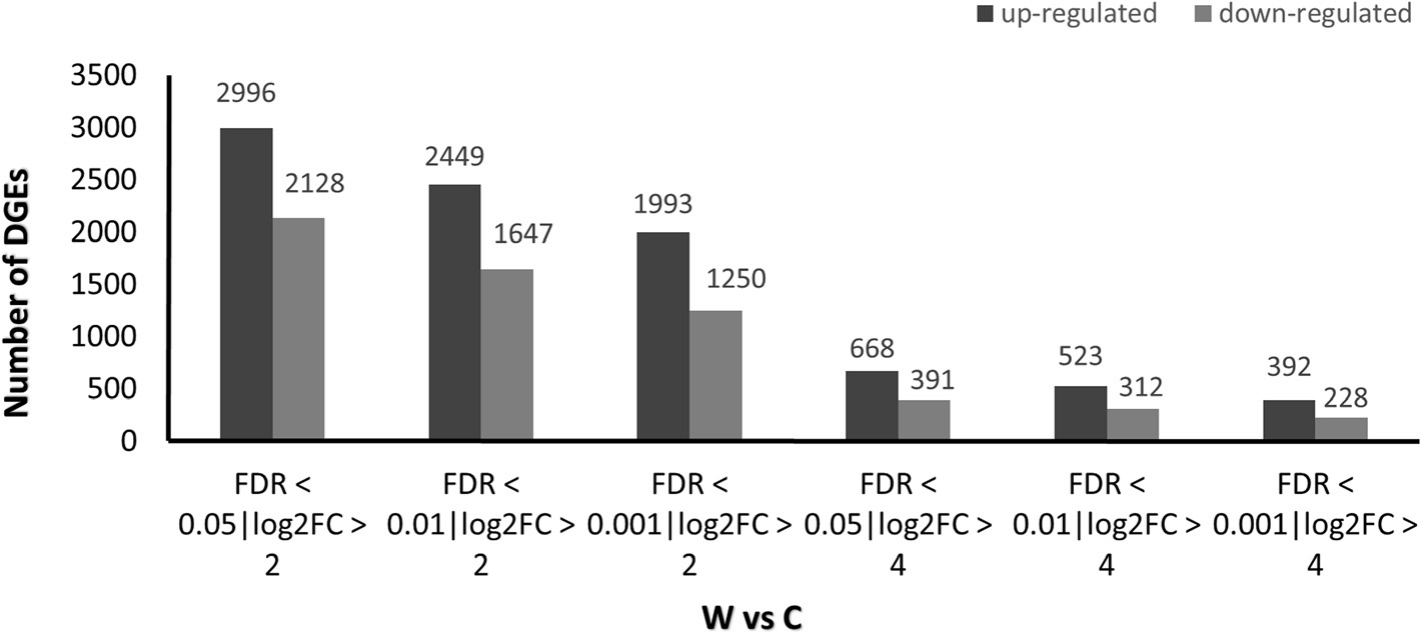
Number of differentially expressed unigenes between the treatment (W) and the control (C) set at different stringencies

Differentially expressed genes between the two libraries (W and C) analysed using edgeR at a cut-off of FDR < 0.05 and log2FC > 2 showed that 2996 transcripts were significantly up-regulated while 2128 transcripts were significantly down-regulated in rubber wood (Fig. 4, Additional file 5: Table S4 and Additional file 6: Table S5). Increasing the stringency of differentially expressed genes progressively up to FDR < 0.001 and log2FC > 4 also reveal a high number of significantly expressed transcripts, 392 up-regulated and 228 down-regulated (Fig. 4). A subset of the most highly up-regulated and down-regulated transcripts with functional annotation are shown in Tables 3 and 4.

**Table 3:** Twenty most up-regulated genes with functional annotation during saprotrophic growth of *R. microporus* on *H. brasiliensis*

Gene ID^a^	log2FC^b^	Hit accession number^c^	Hit description	Pvalue	FDR
CL2561.Contig1	9.854	ref|YP_007374933.1	NADH dehydrogenase subunit 2	7.32E-07	6.38E-06
CL993.Contig4	9.262	gb|EJD04752.1	Alpha-ketoglutarate	2.40E-110	2.08E-106
CL2913.Contig1	9.167	gb|EJD07867.1	Cephalosporin esterase	1.11E-120	1.28E-116
Unigene7085	8.982	ref|YP_003204905.1	Endonuclease	4.55E-06	3.42E-05
CL4886.Contig1	8.858	gb|EJD00032.1	NAD-binding protein	2.68E-102	1.32E-98
Unigene4438	8.453	gb|EJD07869.1	Alpha/beta-hydrolase	5.03E-125	8.69E-121
CL4040.Contig1	8.302	K01046	Triacylglycerol lipase	2.75E-99	1.05E-95
Unigene7106	8.120	ref|YP_007374941.1	DNA-directed DNA polymerase	2.91E-05	1.85E-04
CL3467.Contig1	8.049	gb|EJD01641.1	Acid protease	1.53E-81	2.63E-78
Unigene6801	7.994	sp|Q5B8K7	Histone transcription regulator 3	3.58E-04	1.77E-03
Unigene7086	7.616	ref|YP_003495111.1	Apocytochrome b	5.32E-05	3.20E-04
CL4964.Contig4	7.609	gb|EJD02610.1	Manganese peroxidase 2	1.77E-107	1.22E-103
Unigene7189	7.428	gb|EJC97283.1	Pol poly protein	9.15E-04	4.07E-03
CL2419.Contig1	7.398	ref|YP_003495099.1	Cytochrome oxidase subunit 2	4.03E-04	1.97E-03
Unigene6789	7.317	sp|Q9UR07	Retrotransposable element Tf2	1.14E-03	4.94E-03
Unigene6787	7.259	sp|Q08438	Phosphopantothenoylcysteine decarboxylase	1.27E-03	5.43E-03
Unigene7063	7.192	ref|YP_001504350.1	Ribosomal protein S3	6.12E-04	2.86E-03
Unigene6978	7.101	ref|YP_004376378.1	Hyp16	1.72E-03	7.08E-03
Unigene776	7.062	sp|Q7RX84	Pre-mRNA-splicing factor	1.52E-84	2.92E-81
Unigene7105	7.032	ref|YP_001504344.1	DNA polymerase 2	1.96E-03	7.92E-03

Gene ID^a^ refers to names of the assembled unigenes; Distinct clusters represented with the prefix (CL) represents cluster unigenes; the same cluster contains similar unigenes (>70 % similarity). Distinct singletons represented with the prefix (Unigene) represents unigenes from a single gene. ^b^Binary logarithm of the fold change calculated from the fragments per kilobase per million reads (FPKM). ^c^Corresponds to best hit of NR/SwissProt/KEGG database

**Table 4:** Twenty most down-regulated genes with functional annotation during saprotrophic growth of *R. microporus* on *H. brasiliensis*

Gene ID^a^	log2FC^b^	Hit accession number^c^	Hit description	Pvalue	FDR
Unigene4869	−10.156	sp|P41816	NADPH dehydrogenase 3	5.53E-57	2.85E-54
CL2604.Contig1	−8.677	ref|XP_001835437.2	YjgH family protein	2.44E-19	1.01E-17
Unigene802	−7.744	gb|EIN08964.1	NAD(P)-binding protein	1.05E-132	3.61E-128
CL391.Contig4	−7.689	sp|P54387	NADP-specific glutamate dehydrogenase	3.89E-14	9.36E-13
CL4092.Contig1	−7.345	gb|EIN05560.1	Cytochrome P450	2.85E-43	6.38E-41
CL2382.Contig2	−7.177	gb|EJD01684.1	Fungal hydrophobin	1.38E-27	1.18E-25
Unigene5403	−7.002	ref|XP_003022235.1	PHD finger and BAH domain protein	7.88E-80	1.13E-76
CL144.Contig15	−6.896	K01210	Glucan 1,3-beta-glucosidase	2.71E-06	2.13E-05
CL160.Contig25	−6.798	gb|EJD02081.1	Pkinase-domain-containing protein	5.11E-08	5.44E-07
CL486.Contig2	−6.570	gb|EJC97746.1	Hexose transporter	8.37E-39	1.45E-36
CL354.Contig15	−6.461	gb|EIW56335.1	Cytochrome P450	1.14E-59	7.28E-57
CL1122.Contig7	−6.444	sp|O14351	Uncharacterized oxidoreductase	2.44E-06	1.93E-05
CL144.Contig16	−6.416	K01210	Glucan 1,3-beta-glucosidase	2.73E-04	1.39E-03
CL476.Contig3	−6.389	gb|EJD04564.1	FAD/NAD-binding domain-containing protein	1.20E-05	8.26E-05
CL1122.Contig8	−6.292	sp|P40580	Benzil reductase	7.59E-06	5.45E-05
Unigene5818	−6.283	gb|EKM54599.1	Glycoside hydrolase family 16	1.34E-90	3.31E-87
CL144.Contig11	−6.175	sp|Q5AVZ7	Glucan 1,3-beta-glucosidase	1.10E-03	4.76E-03
CL354.Contig5	−6.145	gb|EIW56335.1	Cytochrome P450	1.94E-25	1.39E-23
CL1413.Contig3	−6.137	gb|EJD01676.1	14-3-3 protein	1.43E-03	6.02E-03
CL4617.Contig2	−6.126	gb|EGR50668.1	N-terminal WSC domain-containing protein	9.82E-97	3.08E-93

Gene ID^a^ refers to names of the assembled unigenes; Distinct clusters represented with the prefix (CL) represents cluster unigenes; the same cluster contains similar unigenes (>70 % similarity). Distinct singletons represented with the prefix (Unigene) represents unigenes from a single gene. ^b^Binary logarithm of the fold change calculated from the fragments per kilobase per million reads (FPKM). ^c^Corresponds to best hit of NR/SwissProt/KEGG database

### Analysis of genes encoding polysaccharide degrading enzymes during saprotrophic growth on rubber wood

**Fig. 5:**
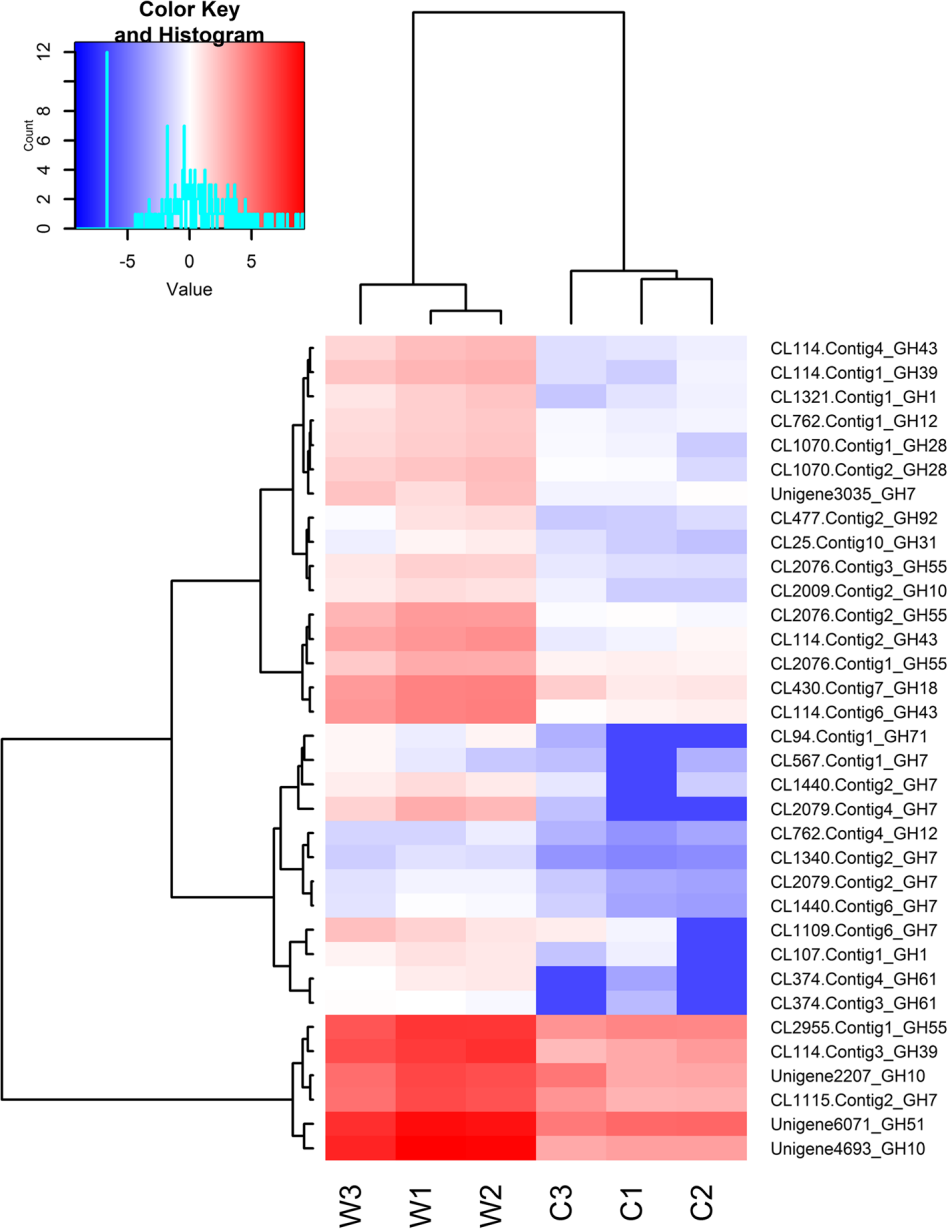
Hierarchical cluster analysis of glycoside hydrolase (GH) family genes up-regulated during saprotrophic growth on rubber wood. (FDR < 0.05 and Fold change > 4). Cluster analysis was constructed based on the log2 values of the fragments per kilobase per million reads (FPKM) of the unigenes. Red indicates high expression and blue indicates low expression

The transcriptome of *R. microporus* produced 173 differentially expressed genes encoding glycoside hydrolases (GH) distributed in 35 families. GH7, GH3, GH15 and GH18 had the highest number of transcripts; 30, 15, 12 and 11 respectively. Altogether, 86 GH genes were up-regulated during growth on rubber wood. All GH12, GH28, GH30, GH35, GH39, GH43, GH51, GH53, GH78, GH79 and GH88 genes were up-regulated in rubber wood (Additional file 7: Table S6, Fig. 5). All GH3, GH17, GH23, GH27, GH37, GH38 and GH72 genes were down-regulated in rubber wood (Additional file 7: Table S6). Seven GH genes; [GH7 (CL2079.Contig4), GH10 (Unigene4693), GH61 (CL374.Contig4), GH71 (CL94.Contig1), GH43 (CL114.Contig6), GH43 (CL114.Contig2), GH61 (CL374.Contig3)] were up-regulated more than 16 fold in rubber wood, with GH7 (CL2079.Contig4) and GH10 (Unigene4693), being up-regulated 71 and 69 fold respectively (Fig. 5, Additional file 7: Table S6). The transcriptome also contained 28 differentially expressed glycosylTransferase (GT) genes distributed in 11 families (Additional file 7: Table S6). The GT1 family gene (Unigene2737) was up-regulated 96 fold in rubber wood (Additional file 7: Table S6, Additional file 8: Figure S2A). Other carbohydrate active enzymes differentially expressed in the transcriptome include 28 carbohydrate esterases (CE) in five families and 7 polysaccharide lyases (PL) in 2 families. Eleven CE genes were up-regulated more than 8 fold in rubber wood, while 4 PL genes were up-regulated more than 4 fold (Additional file 7: Table S6, Additional file 8: Figures S2B and S2C). CE7 (CL2913.Contig1) was up-regulated 575 fold in rubber wood (Additional file 8: Figure S2B) and this gene is among the top 3 most up-regulated genes with functional annotation in the transcriptome (Table 3). Some unigenes involved in glycan biosynthesis and metabolism were also assembled in the transcriptome (Additional file 9: Figure S3).

### Insights on genes encoding glycolignin attacking enzymes during saprotrophic growth on rubber wood

**Fig. 6:**
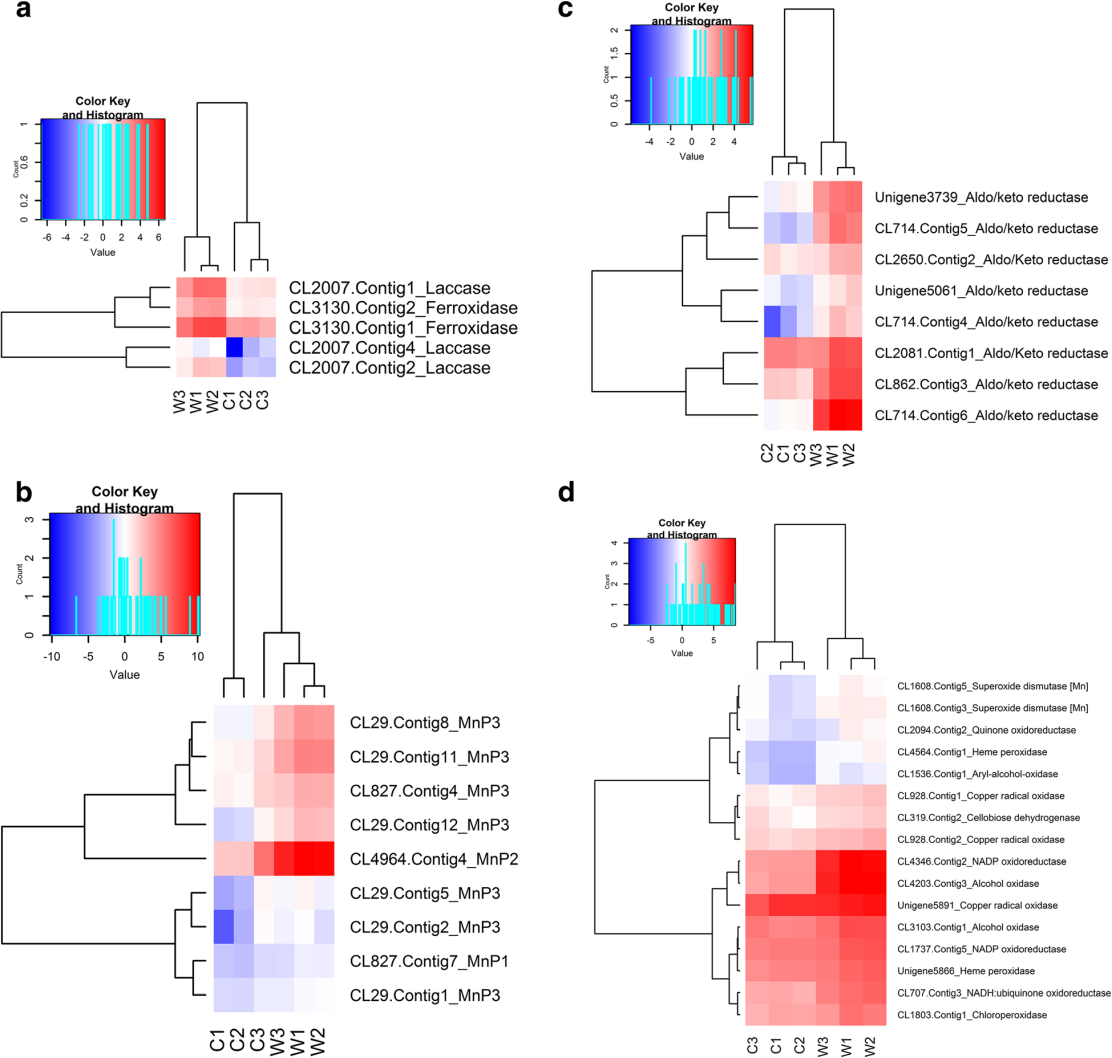
Hierarchical cluster analysis of up-regulated genes involved in lignin degradation during saprotrophic growth on rubber wood. (FDR < 0.05 and Fold change > 2) (**a**) Laccases (**b**) Manganese Peroxidases (**c**) Aldo/Keto reductases (**d**) Cluster of other highly expressed genes. Cluster analysis was constructed based on the log2 values of the fragments per kilobase per million reads (FPKM) of the unigenes. Red indicates high expression and blue indicates low expression

Additionally, 102 genes with capacity for lignin degradation were differentially expressed in the transcriptome. These genes encoding lignolytic enzymes were distributed in 22 families which include multicopper oxidases, class II peroxidases, aldo/keto reductases, alcohol oxidase, copper radical oxidase, superoxide dismutase and NADP oxidoreductase (Additional file 10: Table S7). Nine multicopper oxidases (laccases, ferroxidases) were differentially expressed in the transcriptome, out of which 3 laccases and 2 ferroxidases were up-regulated more than 4 fold in rubber wood (Fig. 6a). Twelve manganese peroxidases (MnP1, MnP2 and MnP3) were differentially expressed with 11 of these up-regulated in rubber wood. All 9 MnP3 genes and the single MnP2 gene in the transcriptome were up-regulated in rubber wood (Fig. 6b, Additional file 10: Table S7). Eight MnP3 genes were up-regulated more than 4-fold while the single MnP2 gene (CL4964.Contig4) was up-regulated 195 fold in rubber wood (Fig. 6b, Additional file 10: Table S7) and is among the top 12 most up-regulated genes with functional annotation in the transcriptome (Table 3). Six aldo/keto reductase genes were up-regulated more than 4 fold in rubber wood (Additional file 10: Table S7, Fig. 6c). An alcohol oxidase (CL4203.Contig3) and NADP oxidoreductase (CL4346.Contig2) genes were up-regulated more than 30 fold in rubber wood (Fig. 6d). A group of 10 yteT-domain oxidoreductase genes were specifically induced only in rubber wood. Five of these genes were up-regulated more than 30 fold (Additional file 10: Table S7, Additional file 11: Figure S4).

### Genes involved in fatty acid and rubber tree latex degradation

KEGG pathway enrichment analysis revealed a number of expressed unigenes in the transcriptome involved in fatty acid biosynthesis, elongation and degradation. Genes involved in natural rubber latex (*cis*-1, 4-isoprene) degradation were also induced in the *R. microporus* transcriptome (Table 5, Additional file 12: Figure S5). Four genes (acyl-CoA synthetase, enoyl-CoA hydratase, 3-hydroxyacyl-CoA dehydrogenase and acyl-CoA acetyltransferase) involved in rubber latex degradation pathway were also detected in the fatty acid metabolism pathways (Table 5). The total number of unigenes in the transcriptome involved in other lipid metabolism pathways is shown in Additional file 12: Figure S5.

**Table 5:** Number of Unigenes expressed and encoding enzymes involved in fatty acid and rubber latex degradation

Pathway	Gene name	No. of unigenes
*Fatty acid biosynthesis*	Acetyl-CoA/propionyl-CoA carboxylase	1
	Acetyl-CoA carboxylase, biotin carboxylase subunit	1
	Fatty acid synthase subunit beta, fungi type	1
	Fatty acid synthase subunit alpha, fungi type	3
	3-oxoacyl-[acyl-carrier protein] reductase	16
*Fatty acid elongation*	3-hydroxyacyl-CoA dehydrogenase	2
	Enoyl-CoA hydratase	1
	Palmitoyl-protein thioesterase	1
*Fatty acid degradation*	Long-chain acyl-CoA synthetase	11
	Acyl-CoA oxidase	1
	Enoyl-CoA hydratase	1
	3-hydroxyacyl-CoA dehydrogenase	2
	Acetyl-CoA C-acetyltransferase	4
	Alcohol dehydrogenase 1/7	5
	Aldehyde dehydrogenase (NAD+)	6
	Unspecific monooxygenase	11
*Rubber tree latex degradation*	Acyl-CoA synthetase	11
	Acyl-CoA dehydrogenase	3
	Enoyl-CoA hydratase	10
	3-hydroxyacyl-CoA dehydrogenase	7
	Acyl-CoA acetyltransferase	8

Unigenes for fatty acid pathways were identified using KEGG pathway enrichment analysis while that of rubber tree latex was compiled manually from the transcriptome data. Rubber tree latex degradation pathway as proposed by Hiessl et al. [49]. Fatty acid biosynthesis/elongation/degradation (FDR < 0.001 and log2FC > 1) and rubber latex degradation (FDR < 0.05 and log2FC > 0.5)

### ATP binding cassette (ABC) transporters and hydrophobins

ATP binding cassette (ABC) transporters belonging to eight families (ABC-A, ABC-B, ABC-C, ABC-D, ABC-E, ABC-F, ABC-G and ABC-I) were expressed in the transcriptome. Two families, ABC-B and ABC-G were more highly expressed. Three transcripts [ABC-G (CL3640.Contig9, CL3640.Contig8); and ABC-B (CL893.Contig27)] were up-regulated more than 5 fold while ABC-G (CL299.Contig2, CL299.Contig1) and ABC-B (CL893.Contig30) were down-regulated more than 9 fold in rubber wood (Additional file 13: Table S8). Several transcripts of hydrophobin encoding genes were differentially expressed during growth on rubber wood (Additional file 14: Table S9). Two hydrophobin genes (CL996.Contig2, Unigene3334) were up-regulated more than 2.5 fold in rubber wood. A fungal hydrophobin (CL2382.Contig2) was down-regulated 145 fold in rubber wood and is among the top 6 most down-regulated genes with functional annotation in the transcriptome (Additional file 14: Table S9, Table 4).

### Analysis of genes encoding enzymes involved in pathways related to energy metabolism

**Fig. 7:**
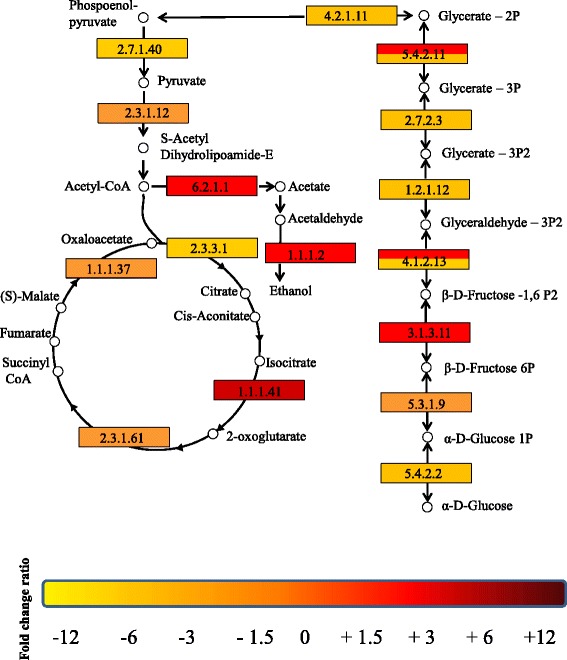
Analysis of pathways related to energy metabolism. The pathway map shows selected steps from KEGG pathways of the Glycolysis/Gluconeogenesis (http://www.kegg.jp/kegg-bin/show_pathway?ko00010) and Citric acid cycle (http://www.kegg.jp/kegg-bin/show_pathway?ko00020). Red indicates up-regulation and yellow, down-regulation in the treatment (W). Boxes with both red and yellow colours indicates cases were some unigenes coding for a particular enzyme were up-regulated while others were down-regulated in treatment (W). The enzymes are indicated with the EC numbers: EC:1.1.1.2, alcohol dehydrogenase (NADP+); EC:1.1.1.37, malate dehydrogenase; EC:1.1.1.41, isocitrate dehydrogenase (NAD+); EC:1.2.1.12, glyceraldehyde 3-phosphate dehydrogenase; EC:2.3.1.12, pyruvate dehydrogenase E2 component (dihydrolipoamide acetyltransferase); EC:2.3.1.61, 2-oxoglutarate dehydrogenase E2 component (dihydrolipoamide succinyltransferase); EC:2.3.3.1, citrate synthase; EC:2.7.1.40, pyruvate kinase; EC:2.7.2.3, phosphoglycerate kinase; EC:3.1.3.11, fructose-1,6-bisphosphatase I; EC:4.2.1.11, enolase; EC:4.1.2.13, fructose-bisphosphate aldolase, class I); EC:5.3.1.9, glucose-6-phosphate isomerase; EC:5.4.2.11, 2,3-bisphosphoglycerate-dependent phosphoglycerate mutase; EC:5.4.2.2, phosphoglucomutase; EC:6.2.1.1, acetyl-CoA synthetase

Analysis of genes in the *R. microporus* transcriptome that encodes enzymes involved in the glycolysis/gluconeogenesis and citric acid (TCA) pathways is depicted in Fig. 7. KEGG pathway enrichment was carried out with a cut-off for significantly expressed genes set at; FDR < 0.001 and Fold Change > 2. Some genes coding for enzymes involved in the early stages of glycolysis, phosphoglucomutase (EC:5.4.2.2) and glucose-6-phosphate isomerase (EC:5.3.1.9) were down-regulated while fructose-1,6-bisphosphatase I (EC:3.1.3.11), was up-regulated in rubber wood. A number of genes involved in the citric acid cycle were also up-regulated in rubber wood; isocitrate dehydrogenase (NAD+) (EC:1.1.1.41), was up-regulated more than 10.5 fold, while alcohol dehydrogenase (NADP+) (EC:1.1.1.2), and acetyl-CoA synthetase (EC:6.2.1.1) were up-regulated more than 3 fold. A summary of the total number of unigenes in the transcriptome involved in other pathways of carbohydrate metabolism is shown in Additional file 15: Figure S6. A global view of the transcriptome by KEGG Gene Ontology (biological process, cellular component and molecular function) enrichment analysis of differentially expressed genes between the two conditions (W and C) is shown in Additional file 16: Figure S7A-C.

### Validation of transcriptome data by qRT-PCR

**Fig. 8:**
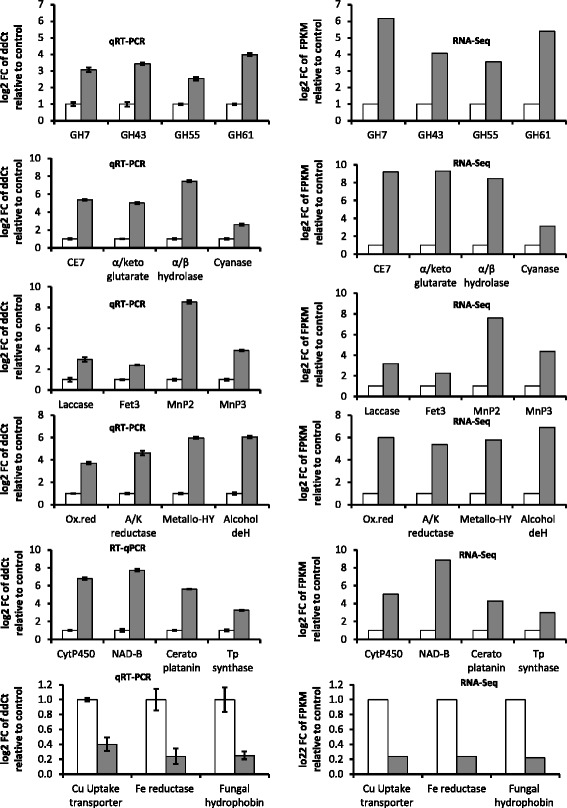
qRT-PCR validation of RNA-Seq expression data. Open bars represent control (C) and closed bars, treatment (W). RNA-Seq relative expression corresponds to log2 fold change (FC) of the fragments per kilobase per million reads (FPKM). qRT-PCR relative expression corresponds to log2 FC of the ddCt values normalized with the reference gene 18S. Bars represent standard error. (Abbreviations: GH, Glycoside hydrolase; CE, Carbohydrate esterase; Fet3, Ferroxidase; MnP, Manganese peroxidase; Ox.red, Oxidoreductase; A/K, Aldo/Keto; HY, Hydrolase; deH, Dehydrogenase; Cyt, Cytochrome; NAD-B, NAD-Binding protein; Tp, Terpenoid). Gene ID of unigenes:[GH7 (CL2079.Contig4), GH43 (CL114. Contig6), GH55 (CL2076.Contig2), GH61 (CL374.Contig4), CE7 (CL2913.Contig1), α/keto glutarate (CL993.Contig4), α/β hydrolase (Unigene4438), Cyanase (CL4402.Contig4), Laccase (CL2007.Contig2), Fet3 (CL3130.Contig1), MnP2 (CL4964.Contig4), MnP3 (CL29.Contig8), Ox.red (CL583.Contig21), A/K reductase (CL714.Contig6), Metallo-HY (CL2357.Contig2), Alcohol deH (CL331.Contig2), CytP450 (CL3949.Contig1), NAD-B (CL4886. Contig1), Cerato platanin (Unigene4679), Tp synthase (CL900.Contig2), Cu Uptake transporter (CL4549.Contig2), Fe reductase (CL1619. Contig4), Fungal hydrophobin (Unigene6195)]

The transcript profiles from the RNA-Seq data was validated by real-time quantitative PCR. Twenty-three genes of interest were selected and the results of the qRT-PCR were compared with the RNA-Seq results (Fig. 8). The qRT-PCR transcript profiles for all the genes tested were consistent with the RNA-Seq data (Fig. 8).

## Discussion

Lignocellulose, the most abundant source of terrestrial carbon and consisting of cellulose, hemicellulose, pectin and lignin is degraded basically by wood and litter decomposing fungi [3, 26]. Members of the white rot fungi belonging to the Polyporales are active wood lignocellulose degraders [27]. *Rigidoporus microporus* is a serious pathogen for rubber plantations in Asia and Africa. The pathogen spreads through root contact and continues to decay wood long after the tree has fallen [1, 2]. It was shown in an earlier study that the isolate used for this transcriptome study caused a dry mass loss of 21 % of *H. brasiliensis* wood blocks after 6 months [28]. Some studies have also shown the ability of the fungus to secret peroxidases and laccases. Previous studies [29, 30] showed that lignin degradation by *R. microporus* involves the synergistic action of manganese peroxidase and laccase, and was enhanced by glucose oxidase. Comparisons of laccase activity from the fungus and other white rot fungi have also been reported [31, 32].

In this study, we performed RNA-Seq *de novo* assembly of *R. microporus* during saprotrophic growth on rubber (*H. brasiliensis* wood) with emphasis on lignocellulose degrading genes employed by the white rot fungus. We also identified potential genes which *R. microporus* could utilize to degrade natural rubber (*cis*-1, 4-isoprene) produced by *H. brasiliensis*, since the fungus is able to survive in the living tree.

In order to generate a high percentage of annotation for the assembled unigenes, five different databases [NR/NT (GenBank) Swiss-Prot, KEGG, COG and GO] were used in the annotation. Analysis of the *de novo* assembly of the transcriptome of *R. microporus* on rubber wood produced 25,880 annotated unigene coding transcripts. In this study, the transcriptome analysis was based on the differentially expressed transcripts between the two conditions, control and saprotrophic growth on rubber wood. The results showed that there was clearly, increased gene expression differences for the transcriptome in rubber wood compared to the control; 2996 transcripts were up-regulated in rubber wood and 2128 transcripts up-regulated in the control. There were over 300 lignocellulose associated transcripts differentially expressed in the transcriptome and the names of the enzymes were confirmed by comparing to the Carbohydrate-Active enZYmes (CAZy) database (http://www.cazy.org).

Glycoside hydrolase family genes encoding for enzymes reported to be involved in cellulose hydrolysis and breakdown were detected in the *R. microporus* transcriptome. GH family genes; GH1, GH7, GH31, GH12, GH55 and GH61 were highly expressed in rubber wood. GH12 and GH55 family genes containing bond cleaving endoglucanases are known to attack β-1, 4-glycosidic bonds in non- crystalline cellulose, while GH1, GH7 and GH31 families act at the ends of cellulose chains [33]. Interestingly, all GH12 transcripts were up-regulated in rubber wood. Crystalline cellulose is degraded mainly by auxiliary activity family (AA9) lytic polysaccharide monooxygenase –LPMO, former GH61 family which is highly induced in our study [34, 35]. Transcriptomic studies also showed up-regulation of GH61 transcripts in wood for *Phanerochaete chrysosporium*, *P. carnosa* and *Heterobasidion annosum* [14, 24, 36]. Relative high transcript levels of genes known to be involved in both crystalline and non-crystalline cellulose were present in the transcriptome. Hemi-cellulose breakdown is more complex because of the presence of acetyl groups and covalent cross-linkages, thus degradation requires several backbone cleaving and de-branching enzymes [3, 37]. GH10, GH39, GH43, GH51, CE1 and CE15 genes were strongly up-regulated in rubber wood and have been implicated in hemicellulose degradation. GH10 and GH11 family genes comprising xylanases are needed for xylan breakdown [38]. Xylosidases from GH3 and GH39 families are involved in degradation of xylooligosaccharides to monosaccharides [39]. GH43 family comprises a variety of enzymes which cleave glycosidic linkages of hemicellulose [15]. Interestingly, all GH39 and GH43 genes in the *R. microporus* transcriptome were up-regulated in rubber wood. Hemicellulose breakdown also involves the actions of GH51 and GH54 (comprising α-arabinofuranosidases), CE1 (acetyl xylan esterases) and CE15 (glucoronoyl esterases) which collectively complete the degradation process by cleaving backbone chains and side groups [40]. Pectin breakdown is carried out by GH28 (rhamnogalactoronases and xylogalactoronase), GH78 (α-rhamnosidases) and GH88 (glucoronyl hydrolases) by cleaving of complex branching [41]. All GH28, GH78 and GH88 transcripts were up-regulated in rubber wood indicating a potential implication in pectin degradation during saprotrophic growth. Moreover, these genes might also be relevant for the pathogenic ability of this fungus, as GH28 family play an important role in pectin degradation in fungal pathogens [42]. CE family genes; chitin deacetylase (CE4) and pectine esterases (CE8) previously identified in genomes of some white rot polypores [27] were found to be highly expressed in the transcriptome. Several putative cephalosporin esterase (CE7) family genes not previously characterized in basidiomycetes were highly expressed in rubber wood. The enzyme cephalosporin esterase can deacetylate various cephalosporins. Cephalosporin belongs to the group of β-lactam antibiotics produced by some fungi and horizontal gene transfer from bacteria to fungi has been proposed as part of its evolutionary origin [43]. The CE7 gene highly expressed in rubber wood share closest similarity (54 %) with the predicted cephalosporin esterase of the white rot fungus, *Fomitiporia mediterranea* (GenBank: EJD07867.1). Further study and characterization of this gene could shed more light on its function in basidiomycetes.

A number of laccases and a large number of manganese peroxidases were highly induced in rubber wood. Laccases together with the class II heme-containing peroxidases (lignin, versatile and manganese peroxidases) are the major enzyme machinery used by white rot basidiomycetes in lignin deconstruction [44]. Manganese peroxidase (MnP) and lignin peroxidase (LiP) are the most effective lignin degrading enzymes with MnPs more widely distributed in basidiomycetes and are currently receiving more attention as potential sources of ligninolytic enzymes [45, 46]. In our study, 5 laccases and 9 MnPs transcripts were significantly up-regulated in rubber wood, which might suggest potential relevance of these enzymes for lignin degradation. The MnP2 gene highly expressed in rubber wood share closest similarity (75 %) with the MnP2 of the white rot fungus, *F. mediterranea* (GenBank: EJD02610.1). The number of significantly expressed MnPs is high when compared to the number expressed in other white rot transcriptomic studies on wood degradation. Microarray transcriptomic studies of *H. annosum* growing on a gradient of bark, heartwood and sapwood showed that a total of 5 MnPs and 3 multicopper oxidases were significantly up-regulated [24]. RNA-Seq transcriptomic studies by using the model *Pycnoporus cinnabarinus* white rot basidiomycete expressed 3 laccases and 1 MnP in birch wood [21]. These differences could be due to certain technical advantages of deep sequencing RNA-Seq technology (higher increased sensitivity, better discrimination of transcripts and ability to detect new gene models) compared to microarray [47, 48]. Other probable reasons could be due to the fact that, the number and variety of enzymes employed in lignocellulose degradation is quite diverse and depends on substrate, lifestyle and fungal species [15].

*R. microporus* produces extensive rhizomorphs in the soil surrounding rubber trees and its characteristic reddish brown basidiocarps are often on the roots and stems of a decaying tree. Natural rubber latex (*cis*-1, 4-isoprene) which flows from the laticifer tubes of the phloem in *H. brasiliensis* is a defence response to stem wounding. This process is exploited for tapping and collection of rubber latex when a cut is made on the bark of the tree. Survival of the pathogen might require the ability to degrade or survive in latex rich environment of the rubber tree. A degradation pathway for the rubber tree latex has been proposed based on studies on the rubber latex degrading bacteria *Gordonia polyisoprenivorans* [49]. A large number of unigenes that are directly implicated in rubber latex degradation pathway were expressed in the *R. microporus* transcriptome. In particular, multiple transcripts coding for the enzymes; acyl-CoA synthetase, enoyl-CoA hydratase and acyl-CoA acetyltransferase were induced in both growth conditions (W and C) used in this study suggesting constitutive expression of some of the genes. The pathway shows that acyl-CoA synthetase converts the organic acids derived from β-oxidation of the rubber latex into acyl-CoA thioester. Enoyl-CoA hydratase is involved in isomerization while acyl-CoA acetyltransferase releases acetyl-CoA into the citric acid cycle [49]. The potential ability for rubber latex degradation *by R. microporus* is further underscored by the induction of several genes in the transcriptome that are involved in fatty acid degradation. This ability might be crucial for the survival of the pathogen on the living *H. brasiliensis* tree during necrotrophic growth. Given the molecular evidence for a possible host jump from other trees to *H. brasiliensis* and the evolution of the pathogen in absence of *H. brasiliensis* [2], *R. microporus* might have acquired genes that can effectively metabolize fatty acid secondary metabolites produced by the tree. The ability to metabolize and degrade fatty acid related secondary metabolites might be responsible for its ability to survive in the tree in the presence of latex.

Our transcriptome analysis also included genes encoding important fungal proteins like ABC transporters and hydrophobins. ATP binding cassette (ABC) transporters have received much research attention in recent years because of their various predicted functions which includes transport of materials across biological membranes [50]. Two ABC transporter sub-families (ABC-B and ABC-G) which are present in genomes of other white rot polypores were induced in rubber wood, although the precise biological functions for most of the ABC transporters still remain unknown. However, there are evidences for potential involvement of fungal ABC transporters in tolerance and resistance to the chemical defense components of conifer trees. A role for a specific ABC-G transporter in the monoterpene resistance has been demonstrated in the pine pathogen *Grosmannia clavigera* [51] and a similar role has been suggested for an ABC-G transporter in the wood-decay fungus *Phlebiopsis gigantea* [22]. Several hydrophobin genes were differentially expressed in the *R. microporus* transcriptome on wood and non-wood media. Hydrophobins are surface active proteins that have been reported to be implicated in different stages of the fungal lifestyles like fruiting body formation, hyphae growth and emergence, pathogenicity factors and biological control mechanism [52, 53]. The number of expressed hydrophobin transcripts (22) is comparable to 16 transcripts expressed in microarray studies of *Heterobasidion sp.* on pine wood [54].

In order to further elucidate the mechanisms involved in wood degradation and utilization of the degradation products by *R. microporus*, we analyzed genes largely affected during carbohydrate metabolism. Phosphoglucomutases and glucose-6-phosphate isomerase, both involved in the final stages of glucose metabolism were down-regulated in rubber wood. The higher induction of these genes in the control might be due to more resource allocation due to the absence of lignocellulosic substrates in the control. In contrast, fructose-1,6-bisphosphatase I involved in the interconversion of glucose phosphate to fructose phosphate, isocitrate dehydrogenase (NAD+) which converts isocitrate to 2-oxoglutarate and acetyl-CoA synthetase which synthesizes acetyl-CoA for the citric acid cycle were highly up-regulated in rubber wood. Finally, genes which encode alcohol dehydrogenase was also documented. Alcohol dehydrogenase facilitate the interconversion between alcohols and aldehydes or ketones with the reduction of nicotinamide adenine dinucleotide (NAD+ to NADH).

## Conclusions

In this study, we present for the first time, the transcriptomic profile of genes expressed by the white rot fungus, *R. microporus* during saprotrophic growth on rubber (*H. brasiliensis*) wood. The *de novo* RNA-Seq assembly and annotation revealed a very good coverage of the transcriptome. The assembled unigenes contained vast amount of genes encoding major lignocellulose degrading enzymes, especially manganese peroxidases. Our results suggest that the fungus has the capacity to degrade both crystalline and non-crystalline cellulose, hemi-cellulose and pectin. Transcriptome analysis also revealed a large number of peroxidases, laccases, aldo-keto reductases, alcohol oxidases, NADP oxidoreductases and copper radical oxidases utilized in lignin degradation of rubber wood. *R. microporus* also expressed numerous genes involved in fatty acid metabolism and breakdown; a feature supported by its ability to express genes involved in natural rubber latex degradation. Pathway enrichment analysis also revealed some enzymes with potential application in biotechnology. High correlation between the differentially expressed gene assessed by qRT-PCR results and the RNA-Seq results also confirm the reliability of RNA-Seq technology. To conclude, the number of annotated unigenes (25,880) and expressed lignocellulose degrading transcripts (338) indicated that *R. microporus* is a necrotrophic/saprotrophic basidiomycete model with vast capacity to breakdown lignocelluloses.

## Methods

### Fungal strain and growth conditions

*R. microporus* (Isolate ED310) used in this study was obtained from the Forest Pathology Laboratory, University of Helsinki, Finland. It was isolated from a diseased *H. brasiliensis* tree from rubber plantations at the Rubber Research Institute, Nigeria in 2012 [2]. Wood blocks (3 × 1 × 0.5 cm) of *H. brasiliensis* NIG801 clone were oven dried at 65 °C to constant weight. Erlenmeyer flasks (100 ml) containing vermiculite (fraction size – 1 mm) and nutrient solution (gl^−1^: NH_4_NO_3_ – 0.6, K_2_HPO_4_ – 0.4, KH_2_PO_4_ – 0.5, MgSO_4_.7H_2_O – 0.4 and glucose – 1.0) in the ratio 1:6 (1 g vermiculite to 6 ml nutrient solution) was prepared. Three wood blocks were placed in one set of flasks, while the other set had no wood. Flasks were stoppered with cotton wool and aluminum foil and autoclaved for 20 mins. Three freshly growing agar plugs (5 mm^2^) of *R. microporus* were inoculated into each flask. Inoculated flasks were placed in an improvised environmental chamber with wet paper towels. Water was added to the paper towels every 2 weeks to maintain chamber humidity at 60-80 %. Samples from three randomly selected flasks for the two sets of treatment; wood (W) and without wood (C) were harvested after 4 months’ incubation and frozen at −80 °C for RNA extraction.

### RNA extraction, cDNA library construction and sequencing

Total RNA was extracted from three biological replicates of *R. microporus* on wood/nutrient media and on nutrient media alone as previously described by [55]. RNA quality and integrity were confirmed using ND-2000 spectrophotometer (Nanodrop technologies) and Agilent 2100 Bioanalyser (Agilent Technologies, Santa Clara, CA). Library construction and sequencing were performed at the Beijing Genome Institute, Hong Kong (www.bgitechsolutions.com). Messenger RNA was extracted from total RNA using oligo (dT) beads and fragmented in fragmentation buffer to get short fragments of 200 bp. Random hexamers was used to synthesize first stand cDNA, followed by addition of dNTPs, RNase and DNA polymerase I to synthesize second strand cDNA. Sequencing adaptors were ligated to fragments which were amplified by PCR. Six cDNA libraries (3 biological replicates for W and 3 for C) were created. The six cDNA libraries were sequenced separately using the Illumina HiSeqTM 2000 sequencing platform.

### Data filtering, *de novo* assembly and annotation

Raw pair-end reads produced from the sequencing platform were filtered to generate clean reads by removing adaptors, reads with unknown nucleotides larger than 5 % and low quality reads. Clean reads from the six samples were used to perform RNA-Seq transcriptome *de novo* assembly using trinity (http://trinityrnaseq.sourceforge.net/) [56] to generate 90 bp paired end reads. Assembled sequences were defined as unigenes. Unigenes were further clustered into gene family by sequence splicing and redundancy removal to acquire non-reductant unigenes. Unigenes are thus divided into two classes; one with prefix CL (Clusters) having unigenes with similarity between them > 70 % and the second class are singletons with prefix, Unigene. Prediction and annotation of all unigenes were done by Blastx alignment (e value < 0.00001) between unigenes and protein databases like NR/NT (GenBank), Swiss-Prot, Kyoto Encyclopedia of Genes and Genomes (KEGG), Cluster of Orthologous Groups (COG) and Gene Ontology (GO). If results of different databases conflict with each other, a priority order of NR, Swiss-Prot, KEGG and COG was followed. Information from blast results were used to extract coding sequence (CDS) from unigene sequences and translate them into peptide sequences. The CDS of transcripts encoding lignocellulose degrading enzymes were used to characterize isoforms in cluster unigenes. Groups of isoform contigs were counted as a single gene. The systematic analysis of metabolic pathways and functions of gene products was performed using the KEGG http://www.genome.jp/kegg/) [57]. Blast2GO (http://www.blast2go.com/b2ghome) [58] was used to provide annotations of unigenes into biological process, cellular component and molecular function ontologies.

### Analysis of differentially expressed genes

Read counts of genes were calculated by the Fragments Per kilobase per Million reads (FPKM) method [59]. This method eliminates the influence of different gene length and sequencing on calculation of gene expression. Calculated gene expression can be directly used to compare gene expression levels between samples. Differential gene expression between the two libraries (W and C) was analyzed using edgeR (Empirical analysis of Digital Gene Expression in R, http://www.bioconductor.org/packages/release/bioc/html/edgeR.html) package [60]. Heatmaps showing hierarchical clustering were produced using the heatmap.2 package in R software [61]. The false discovery rate (FDR) was used to determine the p-value. We set our threshold for significantly expressed genes at FDR < 0.05 and log2Fold Change (FC) > 2. GO and KEGG functional pathway enrichment analysis was done to show the main biochemical and signal transduction pathways of differentially expressed genes (DGEs) by using a FDR ≤ 0.001 as cut-off value for significantly enriched DGEs.

### qRT-PCR validation of RNA-Seq results

Twenty-three genes of interest (both up-regulated and down-regulated in both experimental conditions) were selected for qRT-PCR validation of RNA-Seq results. Primers for each gene were designed by using the Universal Probe Library Assay Design Center (Roche - http://www.roche.com). Information on primers is shown in Additional file 17: Table S10. One μg of total RNA from the two samples (W and C) were reverse transcribed by reverse transcriptase and random hexamers (Thermo Scientific) to synthesize first strand cDNA. The qRT-PCR was run in a LightCycler 480 SYBR Green I Master (Roche). Reaction was carried out in 384-well plates (Roche) in a total volume of 15 μl containing 5.5 μl cDNA, 1 μl (10 μM) of each primers and 7.5 μl of SYBR green master mix. Cycling conditions are as follows: pre-incubation at 95 °C for 5 min, denaturation at 94 °C for 10 s (4.8 °C s − 1), annealing at 60°C for 10 s (2.5 °C s − 1), extension at 72 °C for 10 s (4.8 °C s − 1), 45 cycles of amplification and final extension at 72 °C for 3 min. Three independent biological replicates and two technical replicates were prepared for each sample. Relative gene expression was calculated using the 2^-ΔΔct^ method. The 18S ribosomal transcript level was used as the internal reference for normalization as its expression in the RNA-Seq data was also found to be relatively stable in all samples (Additional file 18: Table S11).

## Availability of supporting data

The data set supporting the results of this article is included within the article and its additional files.

The raw data from the six samples have been submitted separately to the National Center for Biotechnology Information (NCBI) under the accession number SRP062841. The Transcriptome Shotgun Assembly project has been deposited at DDBJ/EMBL/GenBank under the accession GDMN00000000. The version described in this paper is the first version, GDMN01000000.

## Additional files

Additional file 1: Table S1. Percentage of mapping of each sample. (XLSX 10 kb) Additional file 2: Table S2. Summary statistics of functional annotation of *R. microporus* unigenes in public data bases. (DOCX 13 kb) Additional file 3: Table S3. Annotation of all unigenes from the *R. microporus* transcriptome. (XLSX 9519 kb) Additional file 4: Figure S1. Sequence homology of *R. microporus* transcriptome against NCBI non-redundant (NR) database (A) E-value distribution (B) Similarity distribution (C) Species distribution. (TIF 315 kb) Additional file 5: Table S4. All differentially up-regulated genes during saprotrophic growth of *R. microporus*. (XLSX 1547 kb) Additional file 6: Table S5. All differentially down-regulated genes during saprotrophic growth of *R. microporus*. (XLSX 1631 kb) Additional file 7: Table S6. List of all differentially expressed Carbohydrate-active enzymes [Glycoside Hydrolase (GH), Glycosyltransferase (GT), Carbohydrate esterase (CE) and Polysaccharide lyase (PL) families]. (XLSX 24 kb) Additional file 8: Figure S2. A-C. Hierarchical cluster analysis of (A) GlycosylTransferases (GT) (B) Carbohydrate esterases (CE) and (C) Polysaccharide lyases (PL) genes up-regulated during saprotrophic growth on rubber wood. (FDR < 0.05 and Fold change > 2). Cluster analysis was constructed based on the log2 values of the fragments per kilobase per million reads (FPKM) of the unigenes. Red indicates high expression and blue indicates low expression. (ZIP 703 kb) Additional file 9: Figure S3. Summary of unigenes involved in Glycan biosynthesis and metabolism pathways. (TIF 79 kb) Additional file 10: Table S7. List of all differentially expressed genes involved in Lignin degradation. (XLSX 15 kb) Additional file 11: Figure S4. Hierarchical cluster analysis of yteT oxidoreductases genes up–regulated during saprotrophic growth on rubber wood. (FDR < 0.05 and Fold change > 10) Cluster analysis was constructed based on the log2 values of the fragments per kilobase per million reads (FPKM) of the unigenes. Red indicates high expression and blue indicates low expression. (TIFF 1516 kb) Additional file 12: Figure S5. Summary of unigenes involved in Lipid metabolism pathways. (TIF 123 kb) Additional file 13: Table S8. List of all differentially expressed ATP–Binding cassette (ABC) transporters. (XLSX 394 kb) Additional file 14: Table S9. List of all differentially expressed hydrophobin genes. (XLSX 17 kb) Additional file 15: Figure S6. Summary of unigenes involved in Carbohydrate metabolism pathways. (TIF 153 kb) Additional file 16: Figure S7. A–C. KEGG Gene Ontology (GO) enrichment analysis of differentially expressed genes between the two conditions (W and C) showing the main enriched processes in both experimental conditions. (A) Biological process, (B) Cellular component (C) Molecular function. (ZIP 4695 kb) Additional file 17: Table S10. List of Primers used for quantitative real time PCR. E–Efficiency. (DOCX 21 kb) Additional file 18: Table S11. Expression of 18S ribosomal gene (CL60.Contig2) in the RNA–Seq data. (DOCX 13 kb)

## Abbreviations

KEGG: Kyoto Encyclopedia of Genes and Genomes
COG: Cluster of Orthologous Groups
GO: Gene Ontology
ABC: ATP binding cassette
NCBI: National Center for Biotechnology Information
EST: expressed sequence tag
ORF: open reading frame
FDR: false discovery rate
FC: fold change
GH: glycoside hydrolase
GT: glycosylTransferase
CE: carbohydrate esterase
PL: polysaccharide lyase
CAZy: carbohydrate-Active enZYmes
MnP: manganese peroxidase
CDS: coding sequence
FPKM: fragments Per kilobase per Million reads
DGEs: differentially expressed genes
