# Supplementary material for: De novo transcriptomic assembly and profiling of Rigidoporus microporus during saprotrophic growth on rubber wood
Source: BMC Genomics. 2016 Mar 15;17:234. doi: 10.1186/s12864-016-2574-9 (PMC4791870; doi:10.1186/s12864-016-2574-9)
Supplement: Additional file 2: Table S2. — Summary statistics of functional annotation of R. microporus unigenes in public data bases. (DOCX 13 kb) [file 12864_2016_2574_MOESM2_ESM.docx]

**Table S2:** Summary statistics of functional annotation of *R. microporus* unigenes in public data bases

| **Public data base** | **No. of annotated unigenes** |
| --- | --- |
| NR | 25,724 |
| NT | 7,559 |
| Swiss-Prot | 18,209 |
| KEGG | 17,808 |
| COG | 13,977 |
| GO | 8,742 |
| ALL | **25,880** |
