# Supplementary figures and images for: De novo transcriptomic assembly and profiling of Rigidoporus microporus during saprotrophic growth on rubber wood
Source: BMC Genomics. 2016 Mar 15;17:234. doi: 10.1186/s12864-016-2574-9 (PMC4791870; doi:10.1186/s12864-016-2574-9)

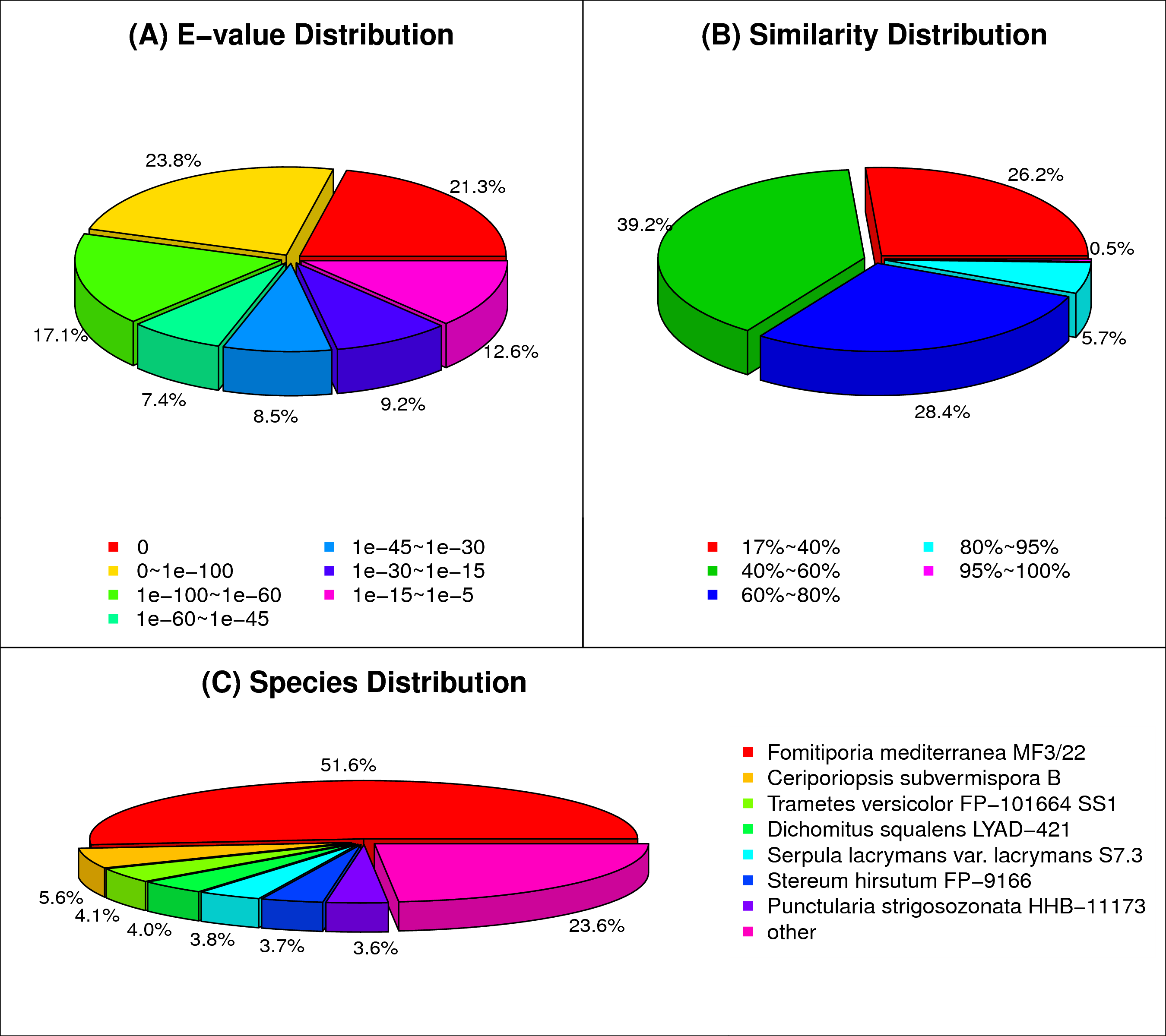

Supplement: Additional file 4: Figure S1. — Sequence homology of R. microporus transcriptome against NCBI non-redundant (NR) database (A) E-value distribution (B) Similarity distribution (C) Species distribution. (TIF 315 kb) [file 12864_2016_2574_MOESM4_ESM.tif]

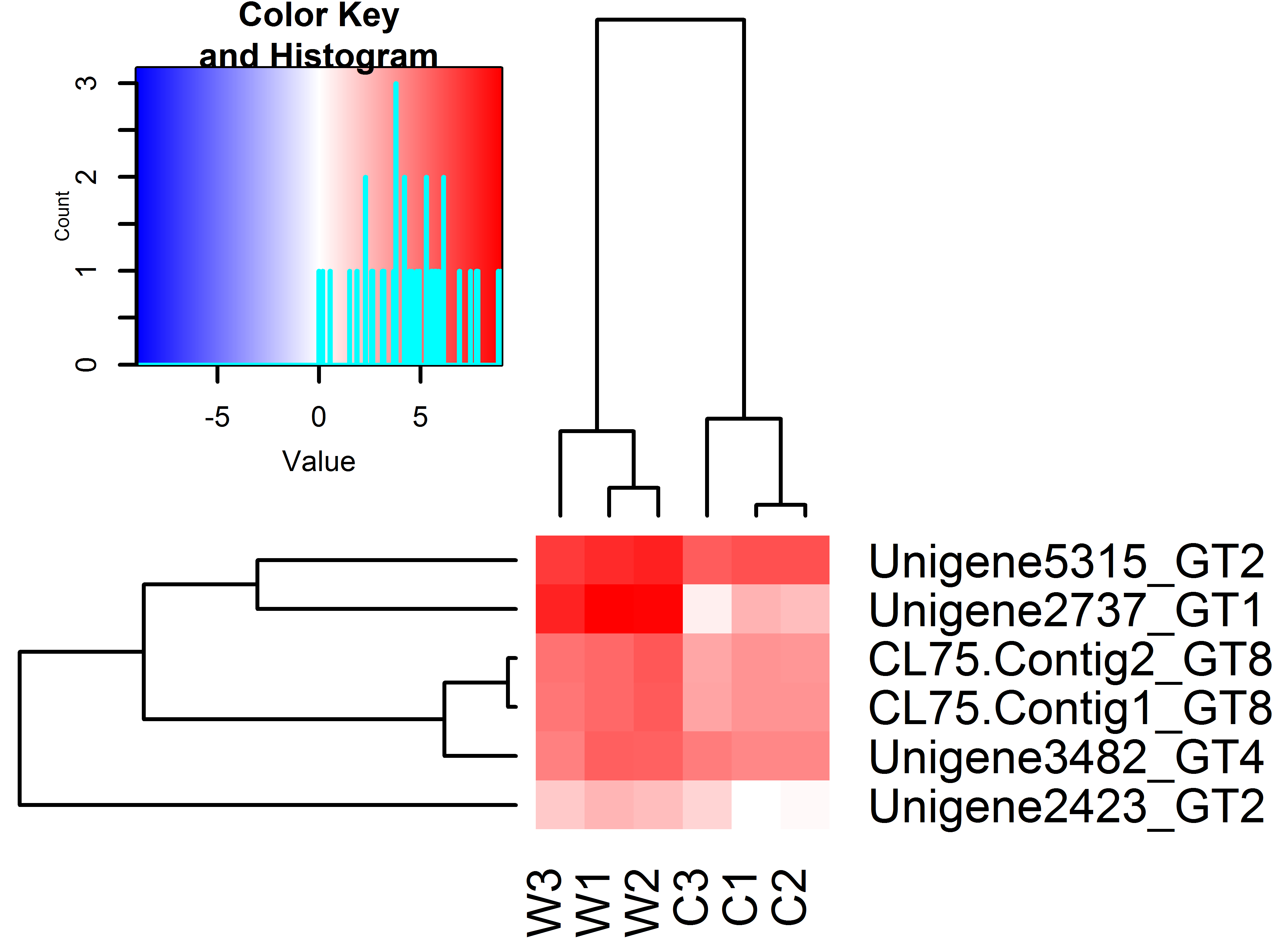

Supplement: Additional file 8: Figure S2. — A-C. Hierarchical cluster analysis of (A) GlycosylTransferases (GT) (B) Carbohydrate esterases (CE) and (C) Polysaccharide lyases (PL) genes up-regulated during saprotrophic growth on rubber wood. (FDR < 0.05 and Fold change > 2). Cluster analysis was constructed based on the log2 values of the fragments per kilobase per million reads (FPKM) of the unigenes. Red indicates high expression and blue indicates low expression. (ZIP 703 kb) [file 12864_2016_2574_MOESM8_ESM.zip › Fig S2/fig S2A.tif]

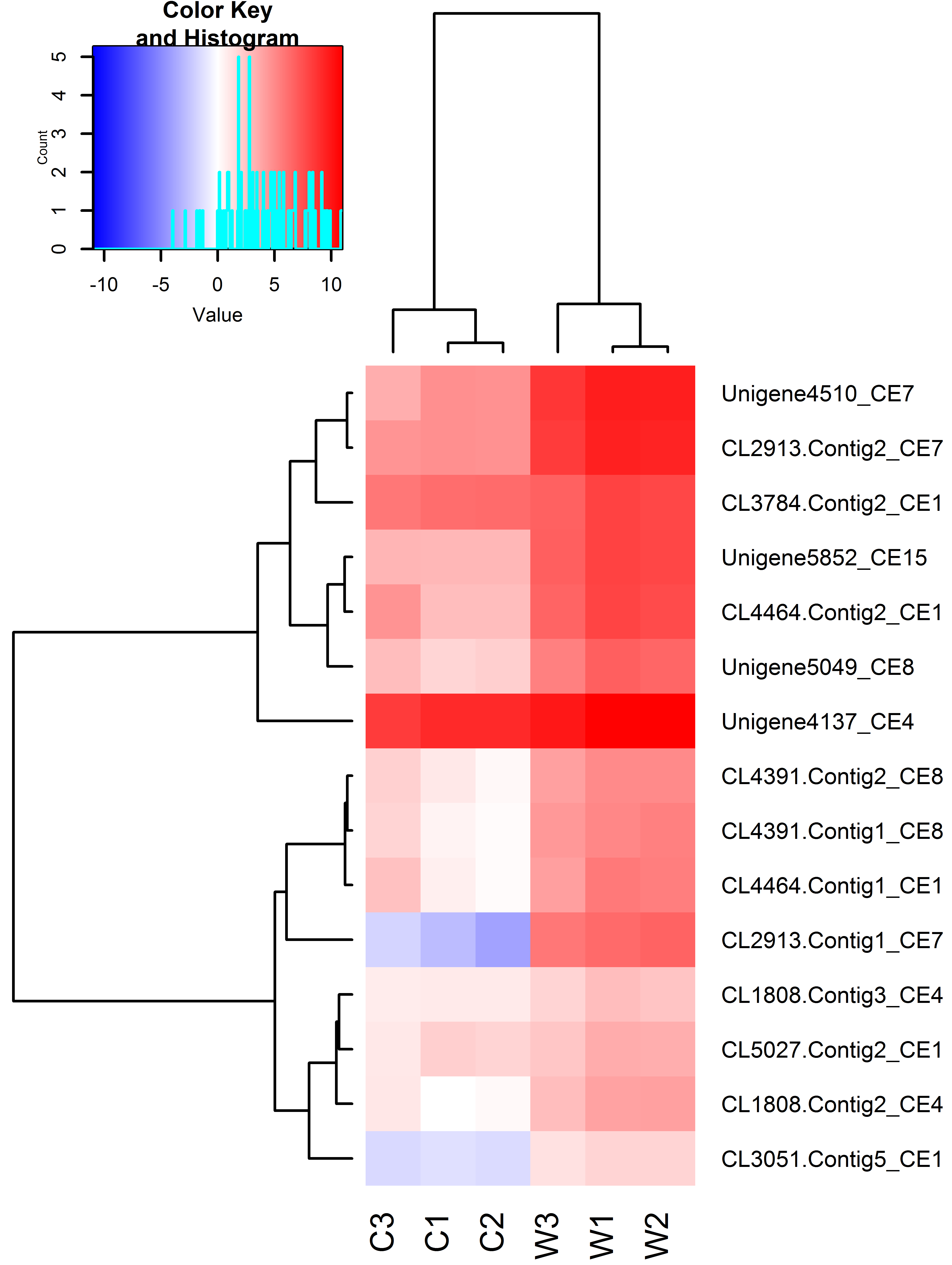

Supplement: Additional file 8: Figure S2. — A-C. Hierarchical cluster analysis of (A) GlycosylTransferases (GT) (B) Carbohydrate esterases (CE) and (C) Polysaccharide lyases (PL) genes up-regulated during saprotrophic growth on rubber wood. (FDR < 0.05 and Fold change > 2). Cluster analysis was constructed based on the log2 values of the fragments per kilobase per million reads (FPKM) of the unigenes. Red indicates high expression and blue indicates low expression. (ZIP 703 kb) [file 12864_2016_2574_MOESM8_ESM.zip › Fig S2/fig S2b.tif]

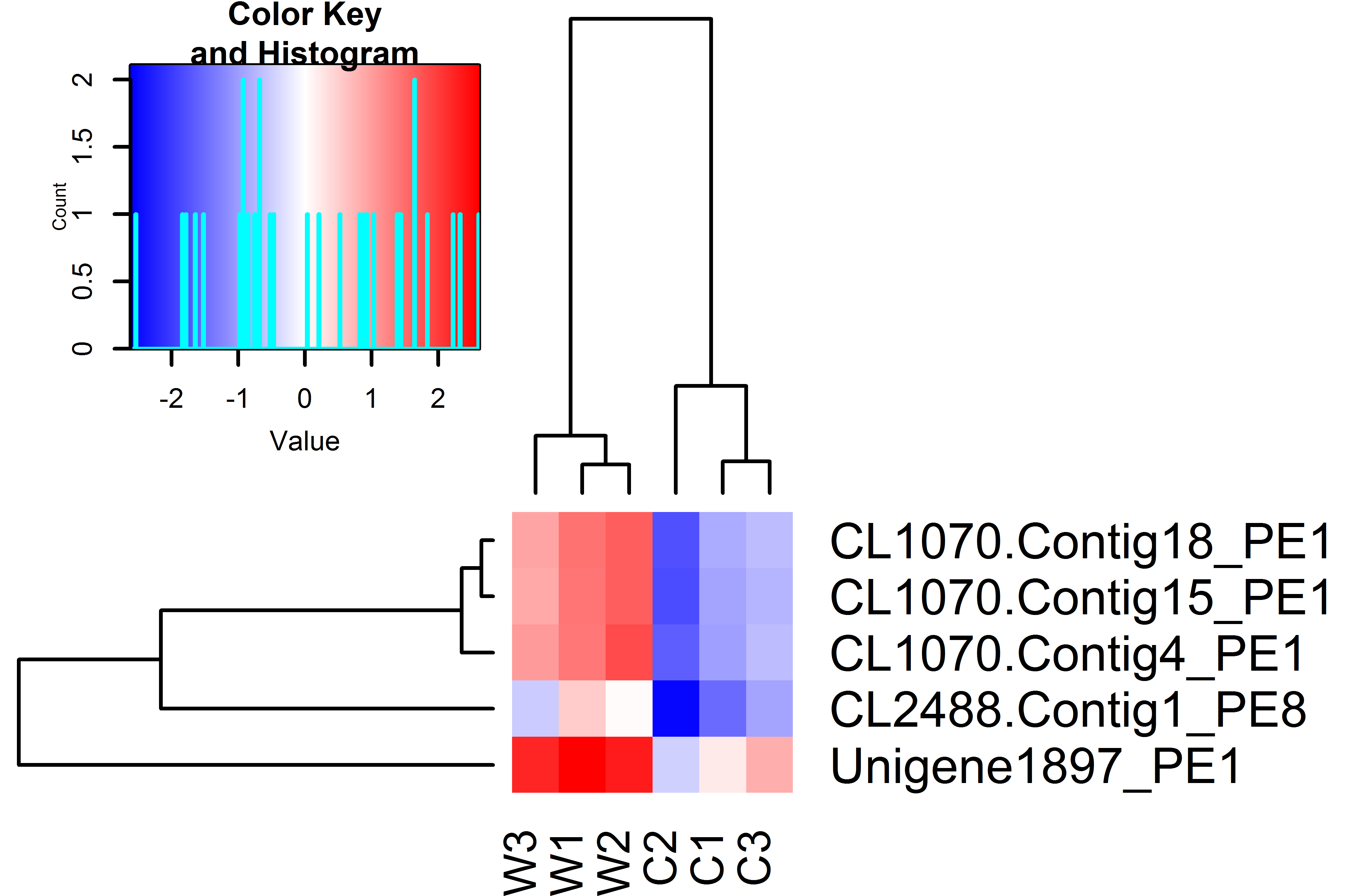

Supplement: Additional file 8: Figure S2. — A-C. Hierarchical cluster analysis of (A) GlycosylTransferases (GT) (B) Carbohydrate esterases (CE) and (C) Polysaccharide lyases (PL) genes up-regulated during saprotrophic growth on rubber wood. (FDR < 0.05 and Fold change > 2). Cluster analysis was constructed based on the log2 values of the fragments per kilobase per million reads (FPKM) of the unigenes. Red indicates high expression and blue indicates low expression. (ZIP 703 kb) [file 12864_2016_2574_MOESM8_ESM.zip › Fig S2/fig S2c.tiff]

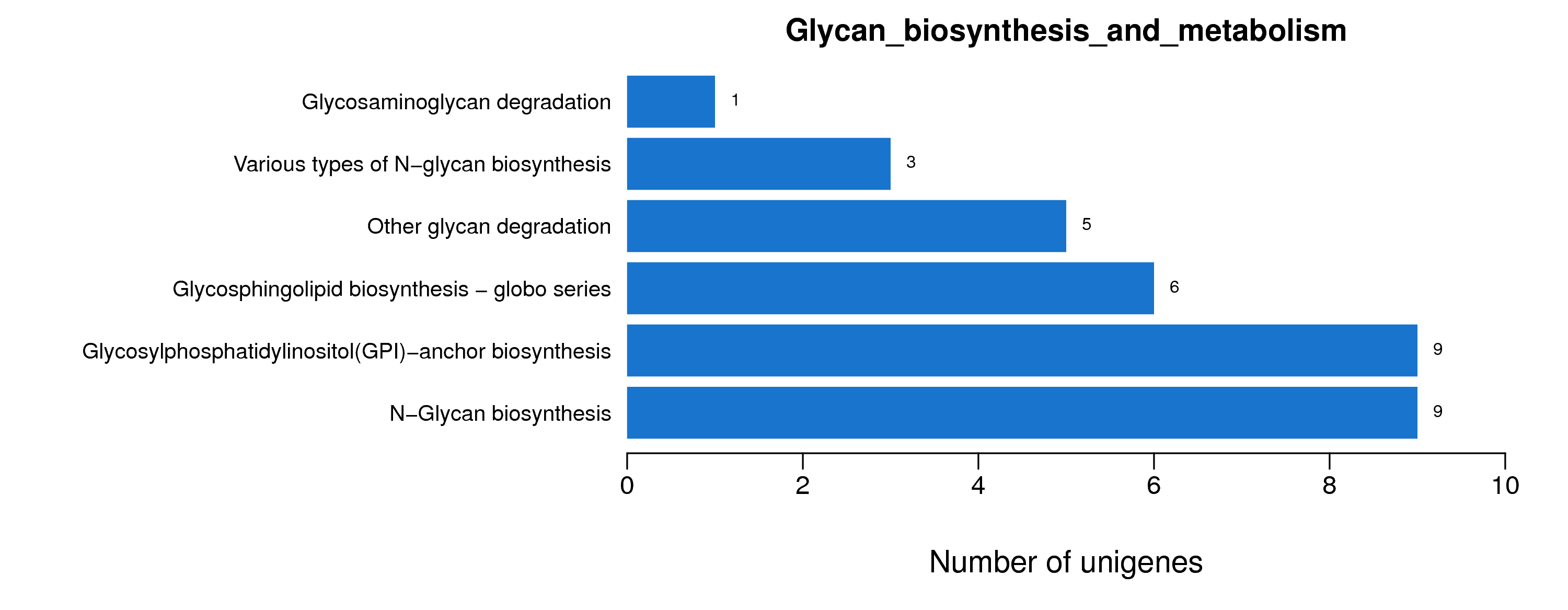

Supplement: Additional file 9: Figure S3. — Summary of unigenes involved in Glycan biosynthesis and metabolism pathways. (TIF 79 kb) [file 12864_2016_2574_MOESM9_ESM.tif]

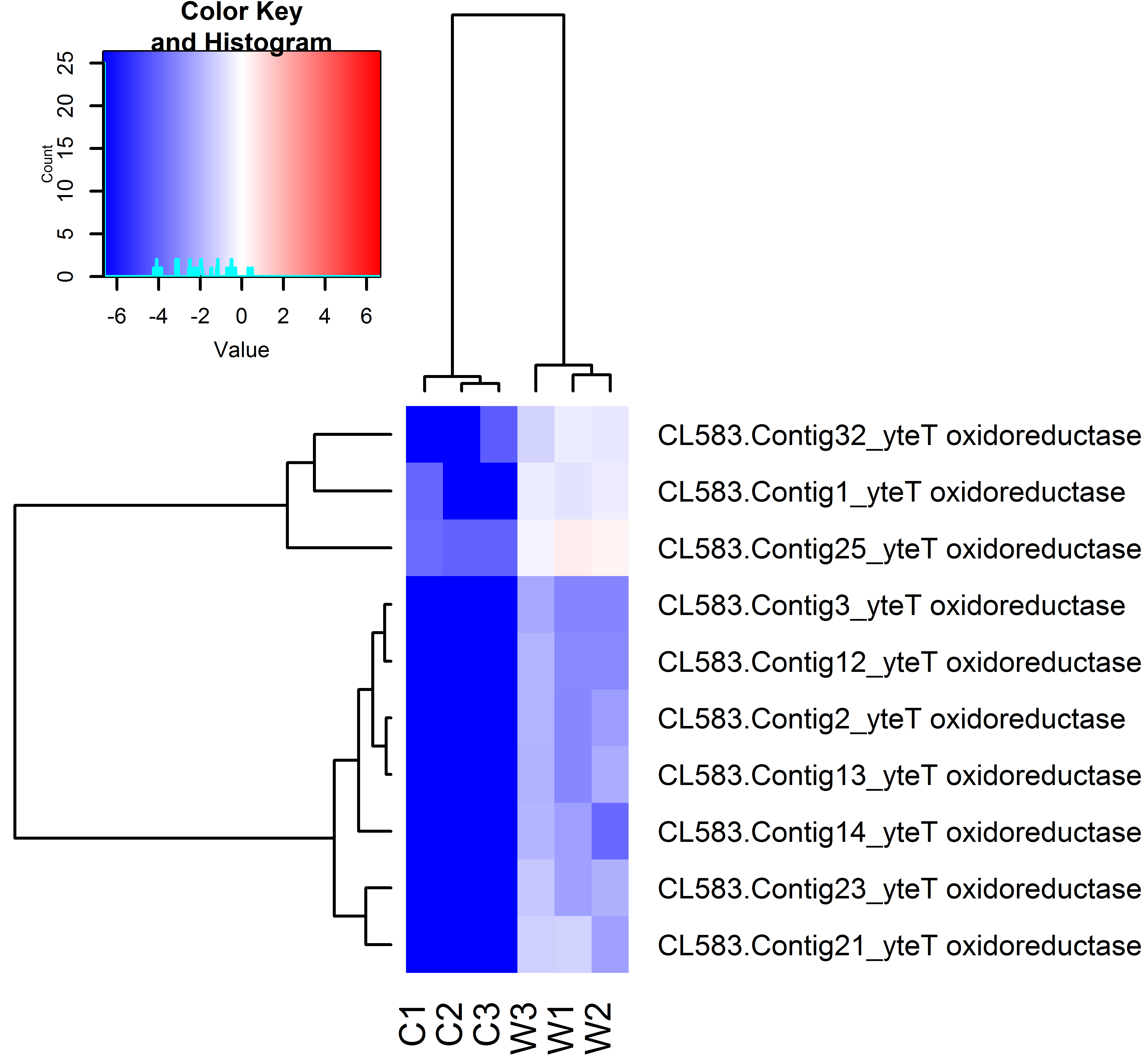

Supplement: Additional file 11: Figure S4. — Hierarchical cluster analysis of yteT oxidoreductases genes up–regulated during saprotrophic growth on rubber wood. (FDR < 0.05 and Fold change > 10) Cluster analysis was constructed based on the log2 values of the fragments per kilobase per million reads (FPKM) of the unigenes. Red indicates high expression and blue indicates low expression. (TIFF 1516 kb) [file 12864_2016_2574_MOESM11_ESM.tiff]

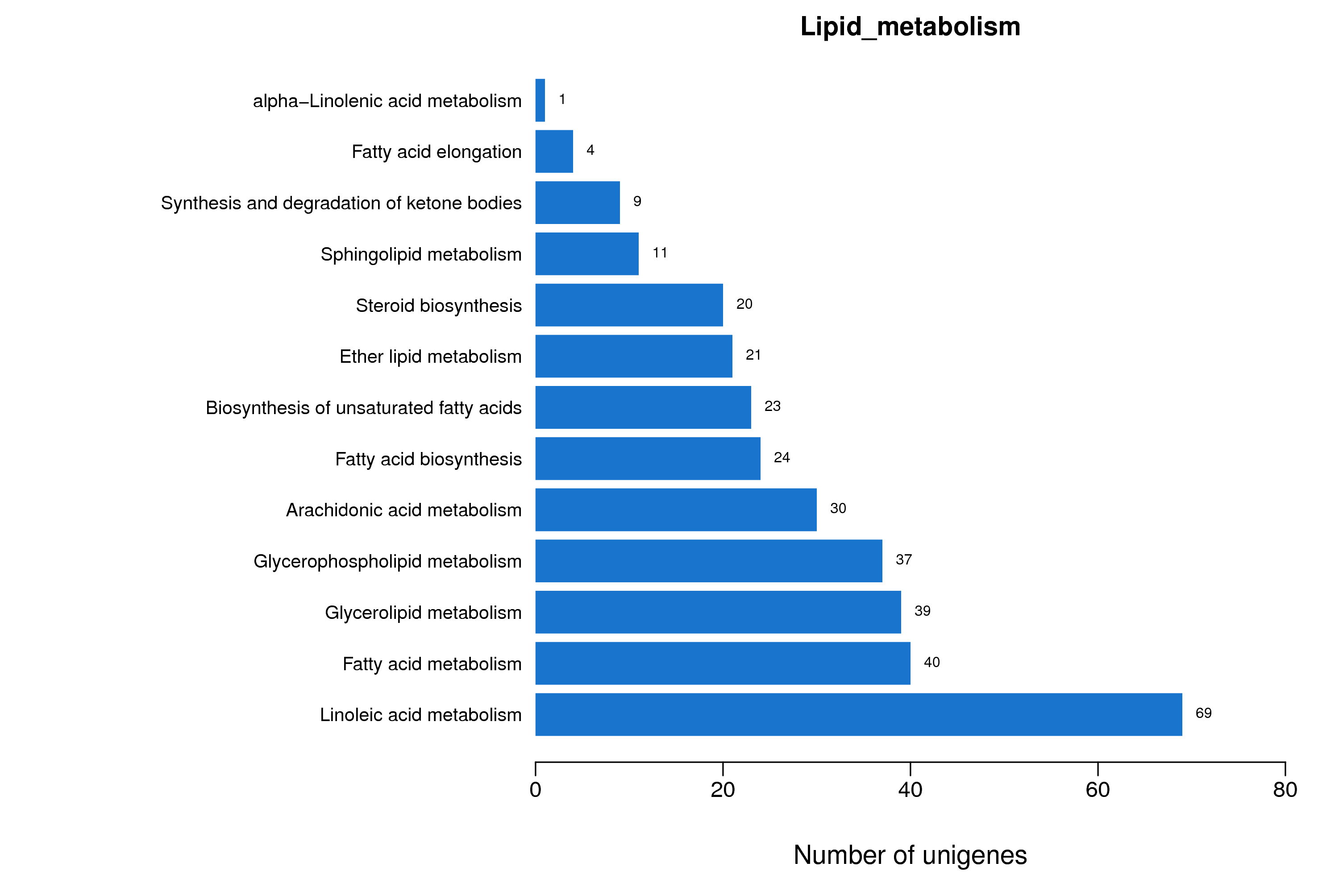

Supplement: Additional file 12: Figure S5. — Summary of unigenes involved in Lipid metabolism pathways. (TIF 123 kb) [file 12864_2016_2574_MOESM12_ESM.tif]

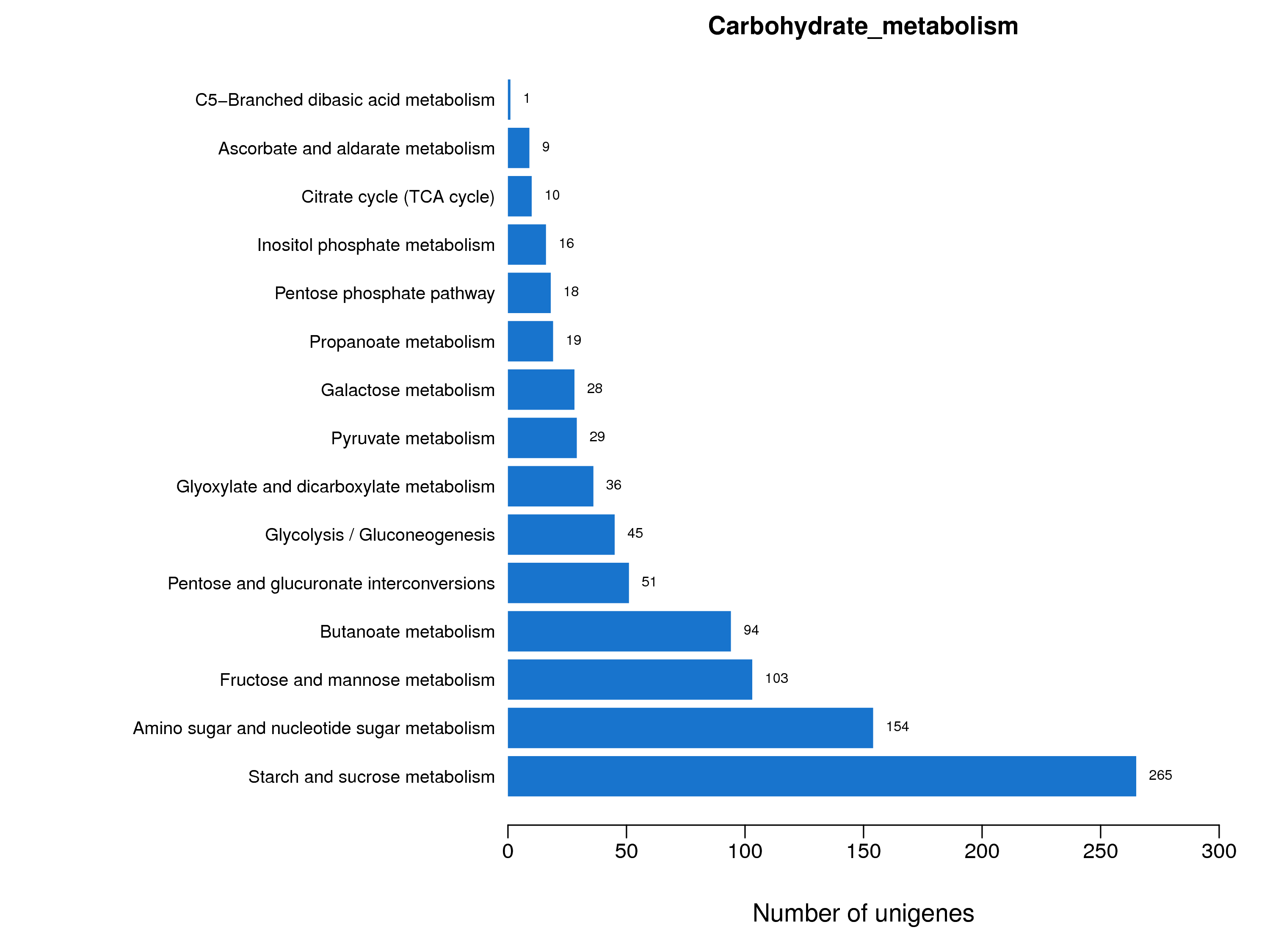

Supplement: Additional file 15: Figure S6. — Summary of unigenes involved in Carbohydrate metabolism pathways. (TIF 153 kb) [file 12864_2016_2574_MOESM15_ESM.tif]

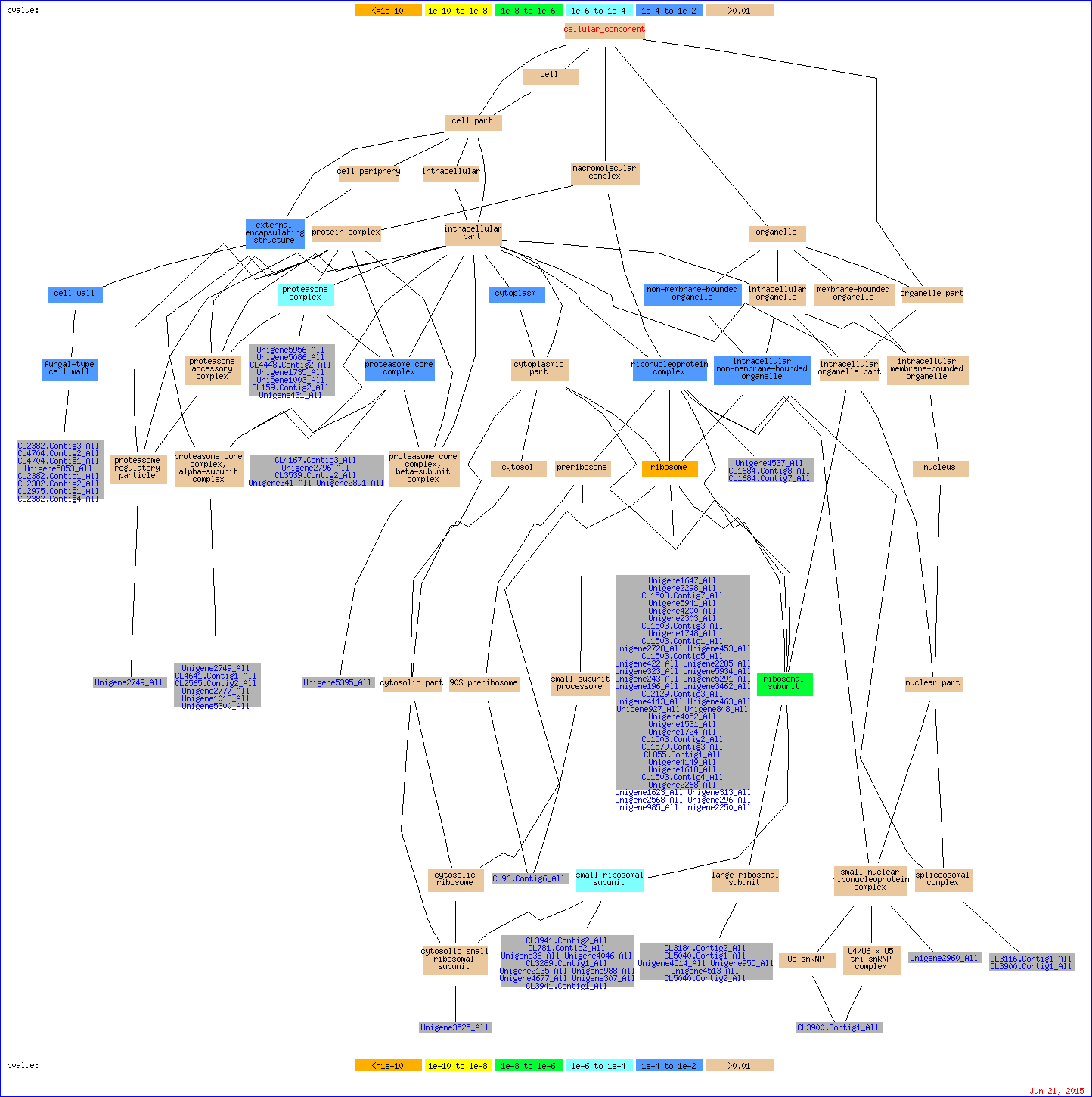

Supplement: Additional file 16: Figure S7. — A–C. KEGG Gene Ontology (GO) enrichment analysis of differentially expressed genes between the two conditions (W and C) showing the main enriched processes in both experimental conditions. (A) Biological process, (B) Cellular component (C) Molecular function. (ZIP 4695 kb) [file 12864_2016_2574_MOESM16_ESM.zip › figS7/fig S7b.tif]

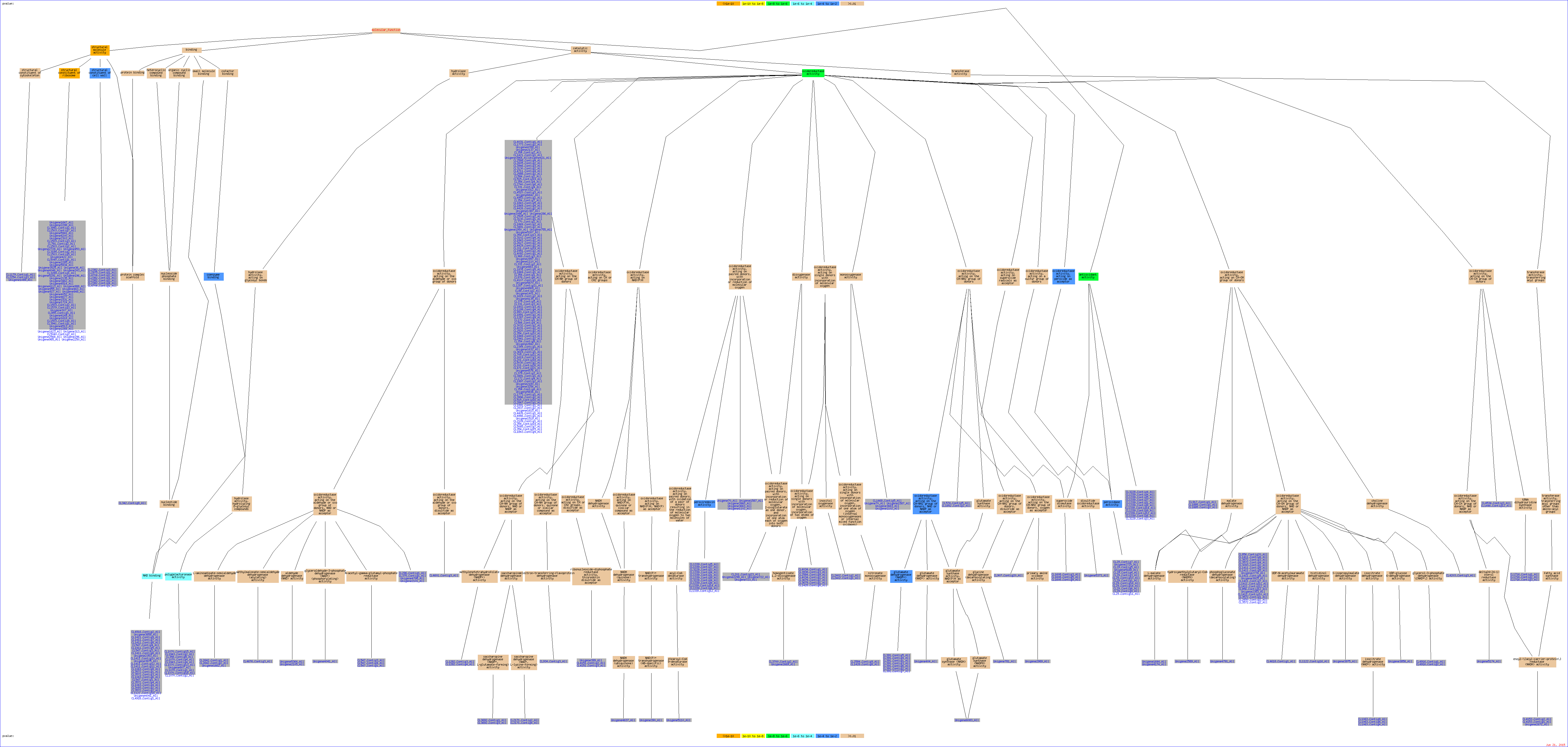

Supplement: Additional file 16: Figure S7. — A–C. KEGG Gene Ontology (GO) enrichment analysis of differentially expressed genes between the two conditions (W and C) showing the main enriched processes in both experimental conditions. (A) Biological process, (B) Cellular component (C) Molecular function. (ZIP 4695 kb) [file 12864_2016_2574_MOESM16_ESM.zip › figS7/fig S7c.tif]
