# Supplementary material for: De novo transcriptomic assembly and profiling of Rigidoporus microporus during saprotrophic growth on rubber wood
Source: BMC Genomics. 2016 Mar 15;17:234. doi: 10.1186/s12864-016-2574-9 (PMC4791870; doi:10.1186/s12864-016-2574-9)
Supplement: Additional file 17: Table S10. — List of Primers used for quantitative real time PCR. E–Efficiency. (DOCX 21 kb) [file 12864_2016_2574_MOESM17_ESM.docx]

**Table S10:** List of Primers used for quantitative real time PCR. E = Efficiency

| **Gene ID** | **Gene Prediction** | **Primers** | **Sequence** | **Amplicon Length** | **E** |
| --- | --- | --- | --- | --- | --- |
| CL2007.Contig2 | Laccase | Forward | TTCCTGCAAACCAGGTTATTG | 65 | 1.86 |
|  |  | Reverse | GTGAAGATGGAATGGGTGGT |  |  |
| CL3130.Contig1 | Ferroxidase | Forward | CGCAATTACGCTTATTGCATC | 66 | 1.98 |
|  |  | Reverse | TGTGGTATCGGAGTTGTTTCTG |  |  |
| CL4964.Contig4 | Manganese peroxidase 2 | Forward | TGGCCAAGATGGCTATTACA | 64 | 1.93 |
|  |  | Reverse | GGGATAACCTCAGAGCAATCA |  |  |
| CL29.Contig8 | Manganese peroxidase 3 | Forward | CCTCTCGATAAACACGCAACT | 67 | 1.92 |
|  |  | Reverse | CTGGACAAGCCTGTTCGAC |  |  |
| CL583.Contig21 | Putative oxidoreductase | Forward | ACGCATGTTGGGCGTATTAT | 60 | 1.96 |
|  |  | Reverse | GCATCCCCTGTGTCTACGTT |  |  |
| CL2079.Contig4 | Glycoside  Hydrolase 7 | Forward | AATATGACGGACCAGGTATTGG | 70 | 1.81 |
|  |  | Reverse | CCATCCAGAAGAACACTGGAA |  |  |
| CL4402.Contig4 | Cyanase | Forward | TCACTGAACTTCACGATGGATT | 72 | 1.89 |
|  |  | Reverse | CTGTGGGTGGTGTTGTTCC |  |  |
| CL2076.Contig2 | Glycoside  Hydrolase 55 | Forward | AACGGAAACGCCTTACTTCC | 63 | 2.12 |
|  |  | Reverse | GCGTCACTCGACGTAAAAGG |  |  |
| Unigene4438 | Alpha/beta hydrolase | Forward | GGACGACTTCACGTTTCCAT | 80 | 1.84 |
|  |  | Reverse | GGATATGGTATGTCCGTTTCCT |  |  |
| CL714.Contig6 | Aldo-Keto reductase | Forward | TCTATCTACACGGGCCAGACA | 63 | 1.87 |
|  |  | Reverse | GCTCATCCGTTGCTTTGAAT |  |  |
| CL114.Contig6 | Glycoside hydrolase 43 | Forward | AGGTGTTGTTGCAGTTGGTG | 65 | 2.05 |
|  |  | Reverse | GAACAACAGCCGAGTCTTCG |  |  |
| CL374.Contig4 | Glycoside hydrolase 61 | Forward | TCGAATACGGCAGAGACGAT | 69 | 1.93 |
|  |  | Reverse | AATTGTCTGATCACACTGGATACC |  |  |
| CL2913.Contig1 | CE7 | Forward | GCTTCAGAGGTTATGGGTGATT | 76 | 1.88 |
|  |  | Reverse | AAGACGCTCTTGGGTAGTGTTC |  |  |
| Unigene4679 | Cerato-platanin | Forward | CCCTAACATAGGCGGGTCTT | 75 | 1.92 |
|  |  | Reverse | AAACCCAAGGTCCAGCAAGT |  |  |
| CL900.Contig2 | Terpenoid synthase | Forward | GGGAAATTGGCACGAGACTA | 70 | 1.96 |
|  |  | Reverse | TGAAACGGGCTTGGACTC |  |  |
| CL993.Contig4 | Alpha-ketoglutarate | Forward | TCACCTGCGTATCATAAACTCG | 73 | 2.08 |
|  |  | Reverse | AGATGTGAGAAGCGATGAACAA |  |  |
| CL2357.Contig2 | Metallo-hydrolase | Forward | GATCGAACACGTCTGCTTGA | 64 | 1.91 |
|  |  | Reverse | CGTGAATGGGTGAGTGTCTC |  |  |
| CL3949.Contig1 | Cytochrome P450 | Forward | AACCTATTATCCGGACCCAAA | 78 | 1.96 |
|  |  | Reverse | TCAGGCTCAGAATCTCTTGCTT |  |  |
| CL4886.Contig1 | NAD-binding protein | Forward | CGACAGTTGATTTCGACCAG | 76 | 1.90 |
|  |  | Reverse | TGTCTCGCTGCGTGCTTAT |  |  |
| CL331.Contig2 | Alcohol dehydrogenase | Forward | AGATGTCGGTTCATATCTCTTCG | 82 | 2.06 |
|  |  | Reverse | CCCAATTGATCGAAGTCCAC |  |  |
| CL4549.Contig2 | Copper Uptake transporter | Forward | GACCTCACTTCGGAAACCAA | 75 | 1.94 |
|  |  | Reverse | TTGGGACTCAACTTGTGGAA |  |  |
| CL1619.Contig4 | Iron reductase | Forward | GGCGTGTGTATGGAAATGG | 72 | 1.95 |
|  |  | Reverse | TGATCTGTCTTTCCATCGATTC |  |  |
| Unigene6195 | Fungal hydrophobin | Forward | TTTTGCCACTTTTGCAAGG | 65 | 1.96 |
|  |  | Reverse | CGTGAGTCCAACTGGAACG |  |  |
| CL60.Contig2 | 18s | Forward | CAGGGTGGTAGACTGTTAAGGAG | 78 | 1.93 |
|  |  | Reverse | GCAACGTAAATGAACTTGGAGA |  |  |
