# Supplementary material for: De novo transcriptomic assembly and profiling of Rigidoporus microporus during saprotrophic growth on rubber wood
Source: BMC Genomics. 2016 Mar 15;17:234. doi: 10.1186/s12864-016-2574-9 (PMC4791870; doi:10.1186/s12864-016-2574-9)
Supplement: Additional file 18: Table S11. — Expression of 18S ribosomal gene (CL60.Contig2) in the RNA–Seq data. (DOCX 13 kb) [file 12864_2016_2574_MOESM18_ESM.docx]

**Table S11:** Expression of 18s ribosomal gene (CL60.Contig2) in the RNA-Seq data

| **Sample** | **No. of Count** | **FPKM** |
| --- | --- | --- |
| W1 | 75 | 2.0076 |
| W2 | 85 | 2.2764 |
| W3 | 68 | 1.3821 |
| C1 | 114 | 3.0599 |
| C2 | 139 | 3.1083 |
| C3 | 206 | 4.5306 |
